# Construction and performance of the millisecond-to-second QXAFS platform at SSRF BL11B for *in situ* dynamic characterization

**DOI:** 10.1107/S1600577526009781

**Published:** 2026-10-29

**Authors:** Yu Chen, Yu Li, Qian Gao, Tao Gan, Shuo Zhang, Jiong Li

**Affiliations:** a Shanghai Synchrotron Radiation Facility, Shanghai Advanced Research Institute, Chinese Academy of Sciences Shanghai201204 People’s Republic of China; b Shanghai Institute of Applied Physics, Chinese Academy of Sciences Shanghai201800 People’s Republic of China; c University of Chinese Academy of Sciences Beijing100049 People’s Republic of China; ESRF – The European Synchrotron, France

**Keywords:** QEXAFS, time-resolved XAFS, synchrotron radiation, *in situ* characterization, noise quantification, energy chemistry

## Abstract

A dedicated quick-scanning X-ray absorption fine-structure (QXAFS) platform has been established at the SSRF BL11B beamline for *in situ* and *operando* studies of dynamic structural evolution on the millisecond-to-second timescale. The system combines a torque-motor-driven Si(111) monochromator, synchronized high-speed acquisition and automated data processing, and operates over an energy range of 5–26.5 keV. Rapid measurements were demonstrated with spectrum acquisition times down to 10 ms for narrow-range XANES, 16.7 ms for full-range EXAFS extending up to *k* ≃ 12.5 Å^−1^, and 50 ms for fluorescence-mode XANES on dilute samples. Quantitative evaluation of frequency-dependent spectral quality further defines practical monochromator oscillation frequencies for the present BL11B platform: approximately 4 Hz for full-range EXAFS over a ∼1000 eV scan window and approximately 30 Hz for short-range XANES over a ∼130 eV scan window, with these recommendations determined by the response characteristics of the current detection system. The capability of the platform is illustrated by *in situ* monitoring of electrocatalyst reconstruction during the oxygen evolution reaction.

## Abstract 2

A millisecond-resolution QXAFS platform has been established at SSRF BL11B for *in situ* and *operando*X-ray absorption studies. The platform demonstrates spectrum acquisition times down to 10 ms for XANES and 16.7 ms for full-range EXAFS, while quantitative analysis of finite-response and statistical limits indicates practically recommended monochromator oscillation frequencies of approximately 4 Hz for full-range EXAFS (∼1000 eV scan window) and approximately 30 Hz for XANES (∼130 eV scan window).

## 1. Introduction

The transition toward a sustainable energy infrastructure relies critically on the advancement of highly efficient energy conversion and storage technologies (Olabi & Abdelkareem, 2022 ▸; Amir *et al.*, 2023 ▸). Consequently, significant progress has been achieved in energy chemistry and catalysis, particularly in electrocatalytic water splitting (Chatenet *et al.*, 2022 ▸; Shih *et al.*, 2022 ▸), electrochemical carbon dioxide reduction (Wang *et al.*, 2021 ▸), and advanced lithium-ion batteries (Xie & Lu, 2020 ▸). The core of these technologies involves complex interfacial reactions driven by the rapid, dynamic evolution of active sites. For example, during the catalytic oxygen evolution reaction, the continuous structural transformations and oxidation state transitions of the catalyst, driven by the applied potential, typically proceed on a timescale of seconds (Vogt & Weckhuysen, 2022 ▸). Similarly, for high-rate battery charging (>10 C), the phase transitions and structural evolution of cathode materials, induced by rapid (de)intercalation, typically unfold on a timescale of seconds to minutes (Zhou *et al.*, 2016 ▸). Accordingly, capturing these *operando* structural dynamics in real time is crucial for unravelling fundamental reaction mechanisms and rationally designing high-performance energy materials.

Among *operando* characterization techniques, quick-scanning X-ray absorption fine structure (QXAFS) enables element-specific electronic states and local coordination changes to be monitored over millisecond-to-minute timescale. For example, sub-second QXAFS has been used to correlate the structural evolution of copper species with product selectivity during pulsed electrochemical CO_2_ reduction (Timoshenko *et al.*, 2022 ▸). In energy storage materials, continuous monitoring of valence and coordination states has been used to follow multistep li­thia­tion and deli­thia­tion pathways of various 3*d* transition metals (Luo *et al.*, 2022 ▸). QXAFS has also been applied to track the structural evolution of single-atom Au species during acetyl­ene hydro­chlorination under realistic working conditions (Malta *et al.*, 2017 ▸). Collectively, these representative *operando* studies demonstrate the utility of time-resolved QXAFS for linking macroscopic functional performance with atomic-level structural evolution.

The continuous-scanning approach underlying QXAFS was developed by Frahm (1988 ▸, 1989 ▸), who originally termed the method quick-EXAFS (QEXAFS) and realized time-resolved XAFS on the timescale of seconds using an ‘on the fly’ mode of a double-crystal monochromator. To further push the limits of temporal resolution, subsequent advancements introduced piezo-actuated monochromators, which dramatically improved the time resolution of XANES to approximately 5 ms (Bornebusch *et al.*, 1999 ▸; Richwin *et al.*, 2001 ▸, 2002 ▸). However, the restricted energy range and practical durability challenges within ultra-high vacuum (UHV) environments hindered the widespread adoption of these piezo-actuated devices. To overcome these constraints, eccentric cam-driven channel-cut monochromators were developed (Frahm *et al.*, 2004 ▸, 2005 ▸). Although mechanically complex, this design combines enhanced long-term stability with a broad energy range, delivering time resolutions down to 12.5 ms (Briois *et al.*, 2016 ▸; La Fontaine *et al.*, 2020 ▸). More recently, direct-drive systems have been developed in which the monochromator crystal is driven directly by a high-speed motor. Nonaka *et al.* reported a direct-drive system based on an AC servo motor, achieving acquisition times of 10 ms for XANES and 25 ms for Cu *K*-edge EXAFS (Nonaka *et al.*, 2012 ▸, 2016 ▸). In a different direct-drive configuration, Müller *et al.* used a torque motor mounted coaxially on a goniometer to drive a rapidly oscillating crystal stage, achieving 10 ms Cu *K*-edge XANES and 16.7 ms Cu *K*-edge EXAFS (Müller *et al.*, 2016 ▸). In these systems, the scan range, oscillation frequency and trajectory can be adjusted through the motor-control parameters. Direct-drive configurations have subsequently been implemented at beamlines including SuperXAS at the Swiss Light Source (SLS), P64 at PETRA III, and beamline 44A at the Taiwan Photon Source (Müller *et al.*, 2016 ▸; Bornmann *et al.*, 2019 ▸; Pao *et al.*, 2021 ▸). Table 1 ▸ summarizes the core characteristics of these QXAFS techniques.

To address the need for tracking rapid structural evolution in energy materials and chemical reactions, we have commissioned a user-oriented QXAFS platform at the BL11B beamline of the Shanghai Synchrotron Radiation Facility (SSRF). This work presents the integration and systematic characterization of the platform, which combines a torque-motor-driven monochromator, synchronized signal acquisition, dedicated detection schemes and automated data-processing software. We describe the beamline implementation and processing workflow, evaluate performance under representative operating conditions, and analyse the factors governing spectral fidelity during rapid acquisition. The capability of the platform is further demonstrated through an *in situ* electrocatalysis experiment, illustrating its applicability to time-resolved studies of dynamic material transformations. Together, these results establish BL11B as a practical QXAFS capability for routine *in situ* and *operando* investigations.

## 2. Experimental section

### 2.1. Beamline overview

**Figure 1:**
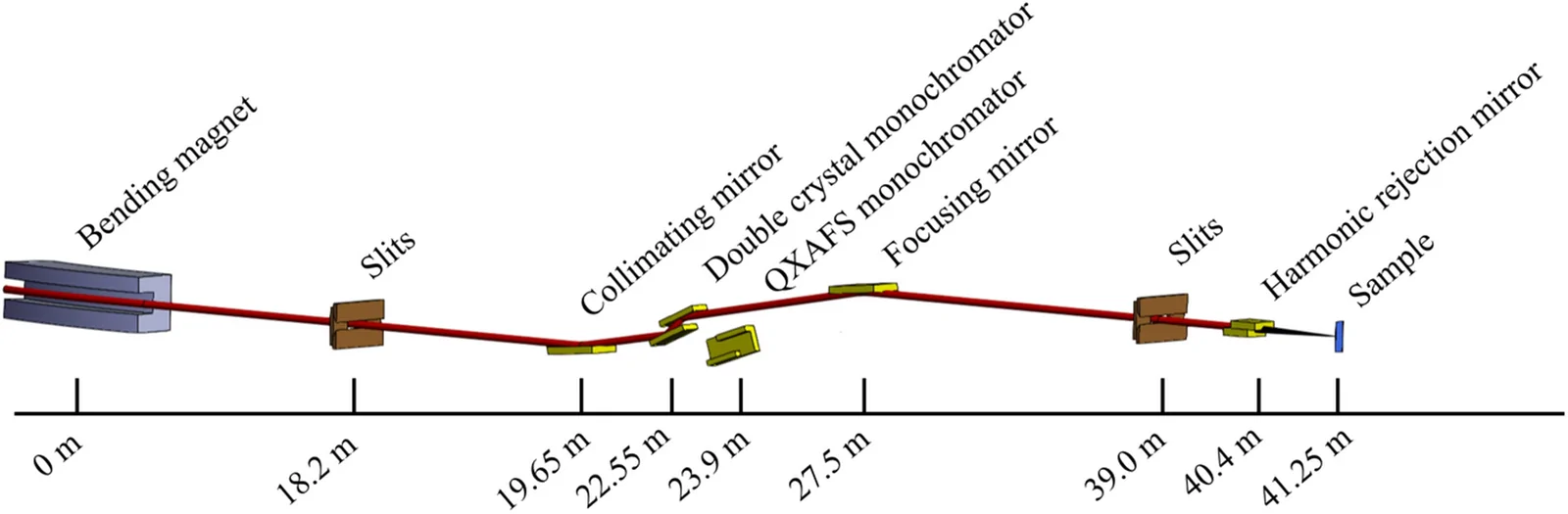
Schematic layout of the main optical configuration at the SSRF BL11B beamline.

The BL11B at SSRF is a bending-magnet beamline dedicated to XAFS studies, designed to address the growing demands of cutting-edge scientific research in fields such as energy materials and catalysis (Chen *et al.*, 2024 ▸). BL11B is the first operational beamline constructed during the SSRF Phase-II project (Tai & Zhao, 2024 ▸). As illustrated in the optical layout (Fig. 1 ▸), the beamline is equipped with a double-crystal monochromator for conventional step-scan XAFS and a dedicated channel-cut monochromator for QXAFS. A Pt/Rh-coated collimating mirror is placed upstream of the monochromator to reduce vertical beam divergence. Downstream, a Pt/Ru-coated toroidal mirror focuses the beam and compensates for the vertical beam offset during XAFS scans. To suppress higher-order harmonics in the low-energy range, a harmonic rejection mirror with Si, Cr, and Rh coatings is positioned upstream of the sample. Primary beamline parameters are summarized in Table 2 ▸.

### 2.2. QXAFS monochromator

**Figure 2:**
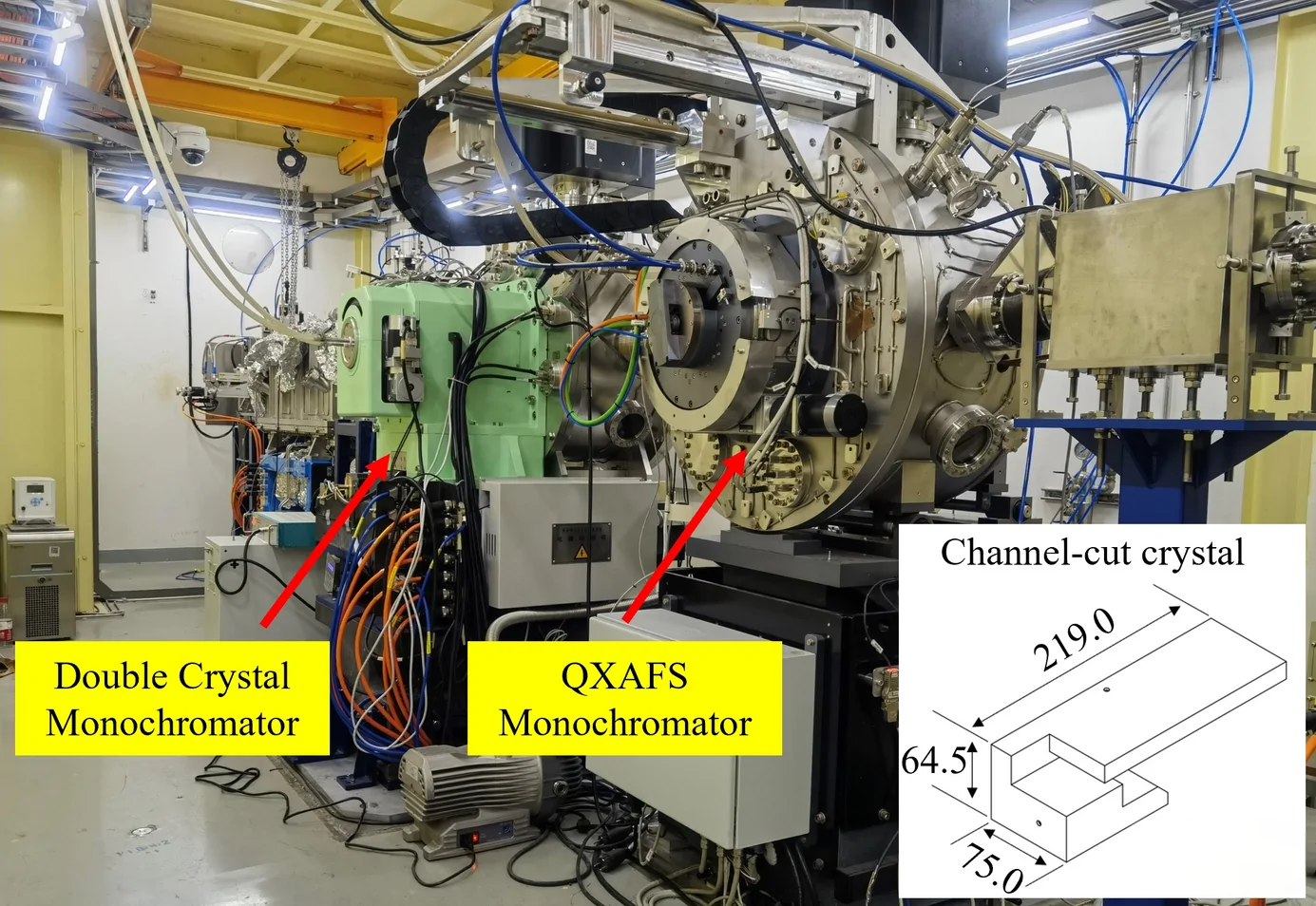
Photograph of the dedicated QXAFS monochromator installed at the BL11B beamline. The inset displays the geometric dimensions of the Si(111) channel-cut crystal (in mm).

The QXAFS monochromator (RI Research Instruments GmbH), located 23.9 m from the source, is the key component for rapid-scanning experiments (Fig. 2 ▸). It employs a torque-motor-driven design originally developed by Frahm (Müller *et al.*, 2015 ▸) and can operate at oscillation frequencies of up to 50 Hz. A Si(111) channel-cut crystal was selected as a practical compromise among photon flux, accessible energy range, energy resolution and time-resolved experimental feasibility. Its usable energy range of 5–26.5 keV covers the *K*-edges of elements from Ti to Ag, matching the primary scientific scope of BL11B, which is focused on *K*-edge XAFS of 3*d* transition metals and selected 4*d* elements.

Although Si(311) offers higher intrinsic energy resolution, Si(111) provides substantially higher photon flux under the present BL11B conditions, typically by a factor of about five to six. This flux advantage is especially important for quick-scanning measurements and for fluorescence-mode studies of dilute or *operando* samples, where photon statistics often become the practical limiting factor. For the intended BL11B applications, Si(111) still provides sufficient spectral fidelity for routine oxidation-state tracking and local coordination analysis, whereas Si(311) remains preferable when the highest possible resolution of subtle near-edge features is required. The spectral performance of the Si(111) configuration near the upper end of the accessible energy range is experimentally evaluated at the Ag *K*-edge in Section S1 of the supporting information.

The Si(111) channel-cut crystal was fabricated with an azimuthal angle of φ = 0°, where φ is defined as the angle between the 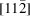 direction and the projection of the electric-field vector **E** onto the (111) surface. To reduce the rotational torque during rapid scanning and minimize beam-position changes over the energy range, the Bragg rotation axis was positioned at the centre of the first reflecting surface of the Si(111) channel-cut crystal. The monolithic crystal block has overall dimensions of 219.0 mm × 75.0 mm × 64.5 mm, with first and second crystal blades of 70 and 167 mm in length, respectively. During QXAFS measurements, the Bragg angle is monitored by an in-vacuum fast incremental optical encoder (Renishaw: Tonic T2651-15M). The encoder and detector signals are recorded synchronously using the manufacturer-supplied QXAFS data-acquisition system described in Section 2.4. The fast-scan encoder provides a theoretical angular resolution of 0.4 µrad (∼0.016 eV at the Cu *K*-edge), far below the experimental sampling grid and the intrinsic core-hole broadening, ensuring that encoder quantization does not convolve or distort the measured line shapes.

### 2.3. QXAFS detection system

For transmission-mode QXAFS measurements, we employed gridded ionization chambers (GICs) with an intrinsic detector response time of less than 5.5 µs (Müller *et al.*, 2013 ▸), providing a response sufficiently fast for the rapid QXAFS measurements reported here. The GICs are operated with nitro­gen gas at atmospheric pressure during the benchmark measurements to maintain consistent detection conditions. For routine user experiments, BL11B is equipped with an automatic He/N_2_/Ar/Kr gas-mixing system, with the gas composition selected according to the absorption-edge of the measured element. Further details are provided in Section S2 of the supporting information.

For fluorescence-yield QXAFS, achieving high temporal resolution requires a high-speed detector for the fluorescence signal. For the millisecond-scale fluorescence QXAFS measurements reported here, the platform utilizes a passivated implanted planar silicon (PIPS) detector with a 50 mm active diameter. The inherent signal ringing, caused by the large junction capacitance of the PIPS diode, was effectively mitigated by integrating a tailored feedback capacitor into the preamplifier circuit (Clark *et al.*, 2020*a* ▸). Systematic tuning of the filter time constants showed that a time constant of 30 µs provides an appropriate balance between signal fidelity and high-speed response. This PIPS-based configuration provided the response required for the millisecond fluorescence QXAFS measurements reported here. For slower *operando* measurements on the sub-second-to-second timescale, a custom multi-grid ionization chamber (fast-Lytle; Lytle *et al.*, 1984 ▸; Suzuki *et al.*, 2002 ▸) was used, as the measured system response (∼650 µs) was adequate for that measurement regime. Detailed detector characterization and application scenarios are provided in Section S3 of the supporting information.

The analog current signals from these detectors, typically in the range of 10^−9^ to 10^−6^ A, are converted to voltage using low-noise, high-speed current amplifiers (ION-4001 and ION-5004, Ionitech Müller & Szillat GbR) and subsequently digitized by a dedicated data acquisition system.

### 2.4. Data acquisition system

The QXAFS monochromator can be operated in two acquisition modes: step-scan and rapid-scan. When operated in step-scan mode, control and data acquisition are handled by the in-house-developed EPICS-based beamline control and data-acquisition system described previously (Lu *et al.*, 2016 ▸; Chen *et al.*, 2024 ▸). For rapid QXAFS measurements, data acquisition is handled by the software supplied with the QXAFS monochromator system by RI Research Instruments GmbH.

This software interfaces with a National Instruments (NI) PXIe-6366 module, which provides eight-channel simultaneous sampling at rates of up to 2 MHz. The Bragg-angle encoder and detector signals are acquired synchronously during continuous monochromator oscillation. The detailed hardware architecture and signal flow are provided in Section S4 of the supporting information.

### 2.5. Graphical data processing software

**Figure 3:**
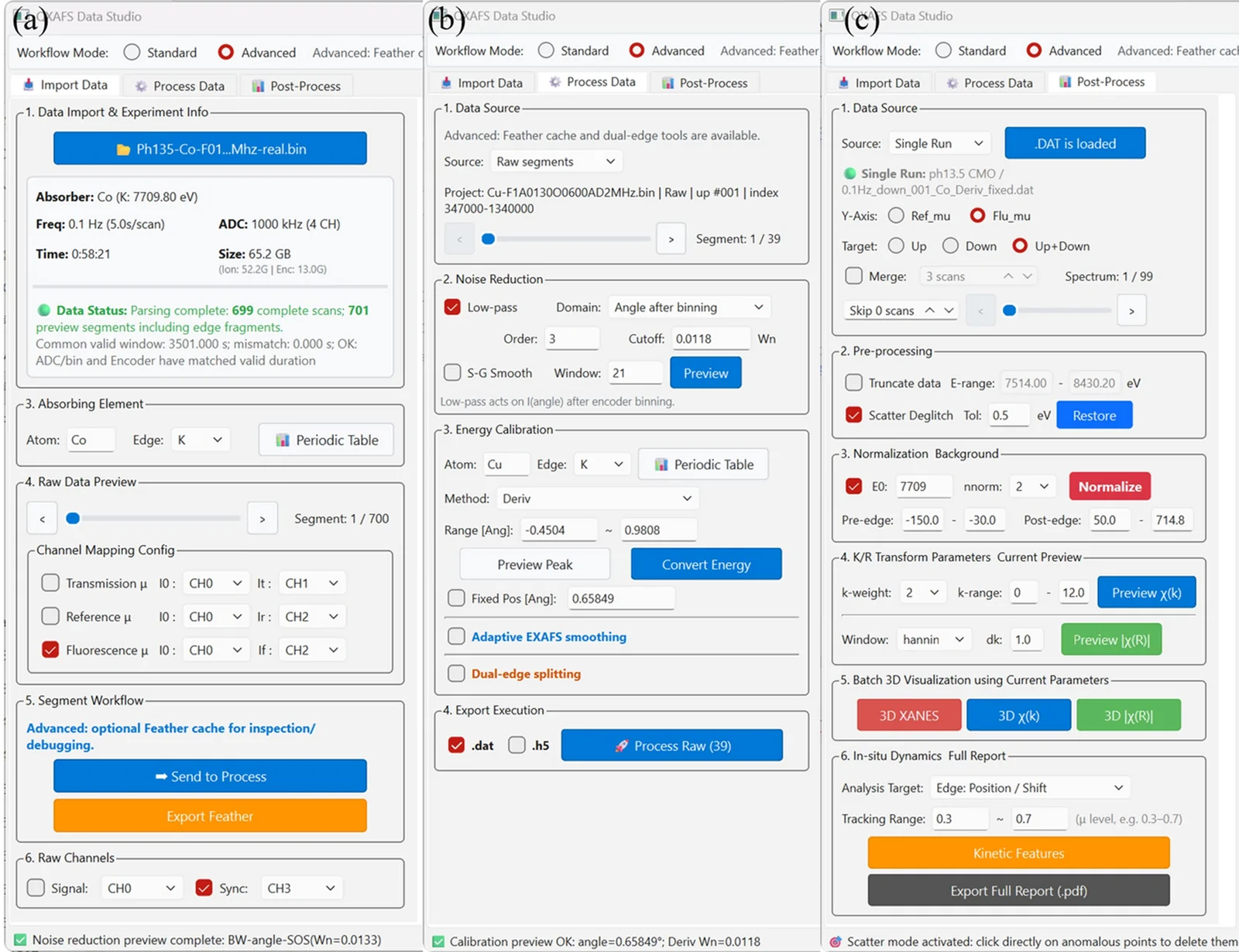
Control panels of the *QXAFS Data Studio* software illustrating the automated three-step workflow. (*a*) Import Data module for binary file reading and automatic sweep splitting based on encoder turning points. (*b*) Process Data module highlighting cascaded noise reduction and the automated batch energy calibration tool. (*c*) Post-Process module featuring interactive background normalization, data transformation, and tools for extracting *in situ* dynamic features.

To efficiently process the high-volume, oversampled QXAFS data streams, we developed a dedicated Python-based graphical user interface (GUI) termed *QXAFS Data Studio*. The software builds on the general processing approach established in *ProQEXAFS* (Clark *et al.*, 2020*b* ▸), while being adapted to the BL11B acquisition architecture and routine user workflow. Designed for the rapid reduction of *in situ* time-resolved datasets, it streamlines the analytical pipeline into three core functional modules (Fig. 3 ▸; see Sections S5 and S6 of the supporting information for details).

(i) Data import and pre-processing. The software rapidly reads binary data files generated by the continuous, high-sampling-rate acquisition of the detectors and Bragg-angle encoder channels. By tracking the encoder signals, it automatically identifies the turning points of the continuous crystal oscillation and separates the decreasing-angle (down) and increasing-angle (up) scans into individual spectra within a single processing run [Fig. 3 ▸(*a*)].

(ii) Data reduction and energy calibration. Addressing the highly oversampled nature of the raw data, this module implements configurable digital filtering—including Butterworth low-pass and Savitzky–Golay filters—to suppress high-frequency noise while preserving the principal spectral features. To convert the measured angular coordinate to photon energy, a fixed energy offset derived from a reference-foil measurement can be applied consistently to all spectra acquired under the corresponding monochromator settings [Fig. 3 ▸(*b*)].

(iii) Post-processing and dynamic analysis. The final module provides a comprehensive toolkit for interactive deglitching, background subtraction, normalization, and standard EXAFS *k*- and *R*-space transformations. Specifically designed for time-resolved studies, it features a 3D visualization suite to map the dynamic evolution of the XANES, χ(*k*), and Fourier-transformed spectra across the entire reaction timeframe. An integrated full-report export function automates the extraction of key spectral parameters (*e.g.* white-line energy position and intensity), generating exportable reports that directly correlate spectral changes with the *in situ* reaction progress [Fig. 3 ▸(*c*)].

### 2.6. Energy calibration

**Figure 4:**
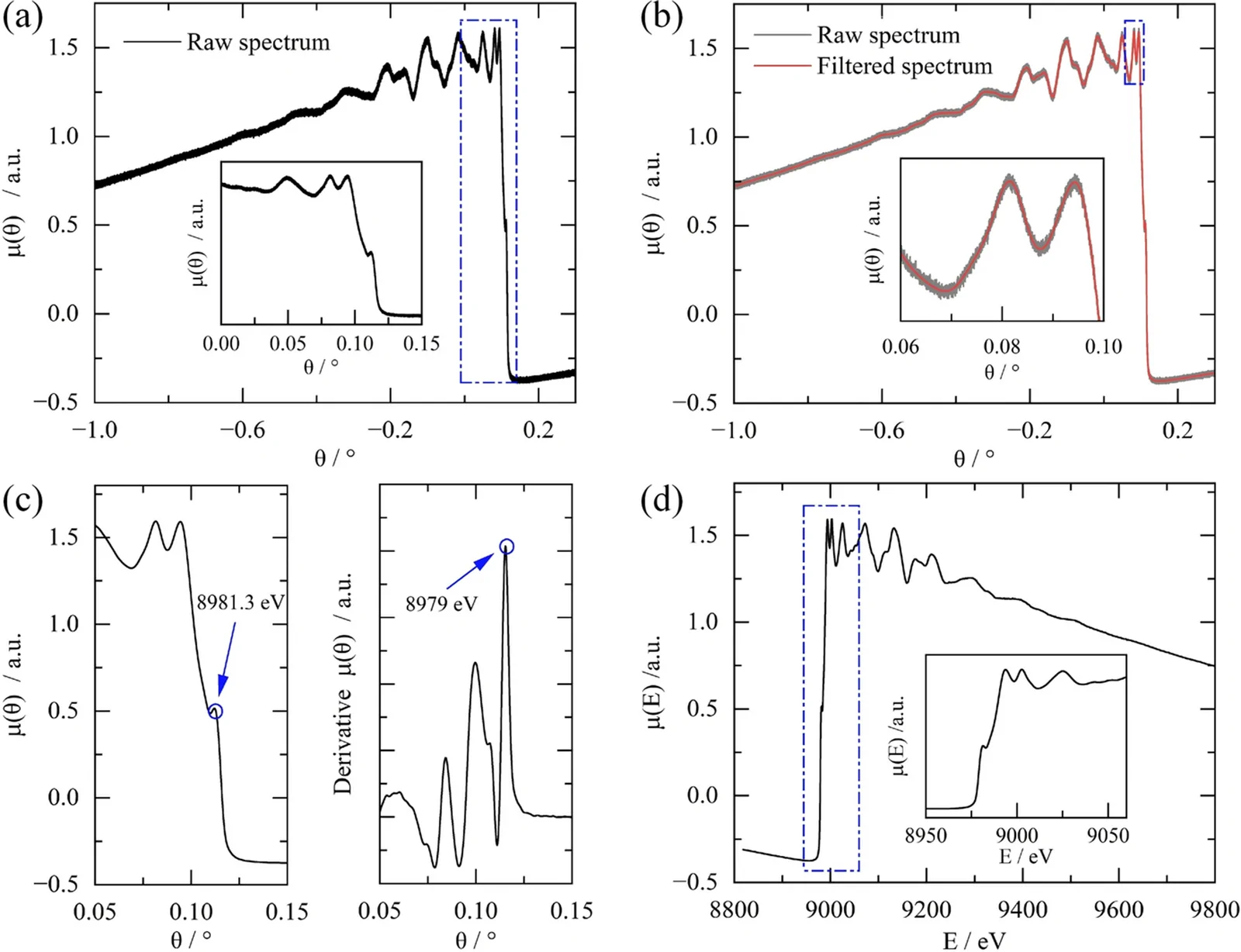
Processing workflow for a single Cu *K*-edge QXAFS spectrum implemented in *QXAFS Data Studio*. (*a*) Raw spectrum extracted from the continuous Bragg encoder trace. (*b*) Comparison of the raw spectrum (grey) and the filtered spectrum (red) as a function of Bragg angle. The enlarged inset shows that point-to-point high-frequency fluctuations are suppressed while the broader spectral profile remains essentially unchanged. (*c*) Extraction of energy calibration references for transmission-mode measurements, showing the characteristic peak alignment (left) and the first-derivative maximum alignment (right). (*d*) The final energy-calibrated XAFS spectrum μ(*E*). The measurements were performed using a Cu foil with a thickness of 4 µm.

The automated workflow of *QXAFS Data Studio* is built upon a streamlined data reduction pipeline, illustrated using a representative Cu *K*-edge spectrum in Fig. 4 ▸. The highly oversampled raw data [Fig. 4 ▸(*a*)] are first reduced by binning detector signal intensities that share identical Bragg encoder readouts. This angle-binned signal is subsequently processed through a zero-phase Butterworth low-pass filter with user-adjustable filter order and cutoff parameter to suppress high-frequency noise [Fig. 4 ▸(*b*); see Section S6 of the supporting information for mathematical details]. Following this noise reduction, precise energy calibration is performed.

In transmission mode, energy calibration is conventionally achieved by simultaneously measuring a reference foil placed downstream of the sample. The Bragg encoder value (θ) is converted to photon energy (*E*) by aligning a characteristic feature of the reference spectrum [*e.g.* the white-line maximum or the first derivative peak of the absorption edge, Fig. 4 ▸(*c*)] with a tabulated reference value (*E*_ref_) using the modified Bragg equation,
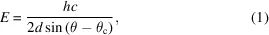
where θ_c_ represents the angular offset extracted from the reference foil measurement. This procedure directly yields the energy-calibrated EXAFS spectrum [Fig. 4 ▸(*d*)].

In many *in situ* fluorescence experiments, the severe X-ray attenuation by the reaction cell precludes simultaneous reference measurements. A conventional approach involves measuring a reference foil mounted on the cell window either before or after the experiment (Rüscher *et al.*, 2022 ▸; Timoshenko *et al.*, 2022 ▸). In the present work, the energy axis was calculated from the Bragg-angle encoder signal and calibrated using a fixed offset determined from a reference-foil measurement acquired under the same monochromator settings. The same offset was subsequently applied to all spectra within the corresponding time series, without spectrum-by-spectrum realignment. This procedure maintains a consistent energy scale while preserving actual sample-induced edge shifts.

## 3. Performance evaluation and rapid-scan spectral characteristics of the QXAFS platform

This section evaluates the QXAFS platform under practical experimental conditions. It first summarizes the main performance metrics of the system, including the accessible energy range and representative measurements in different detection modes, and then examines scan-direction- and frequency-dependent spectral deviations observed during rapid acquisition.

### 3.1. Platform performance

#### 3.1.1. Energy range

**Figure 5:**
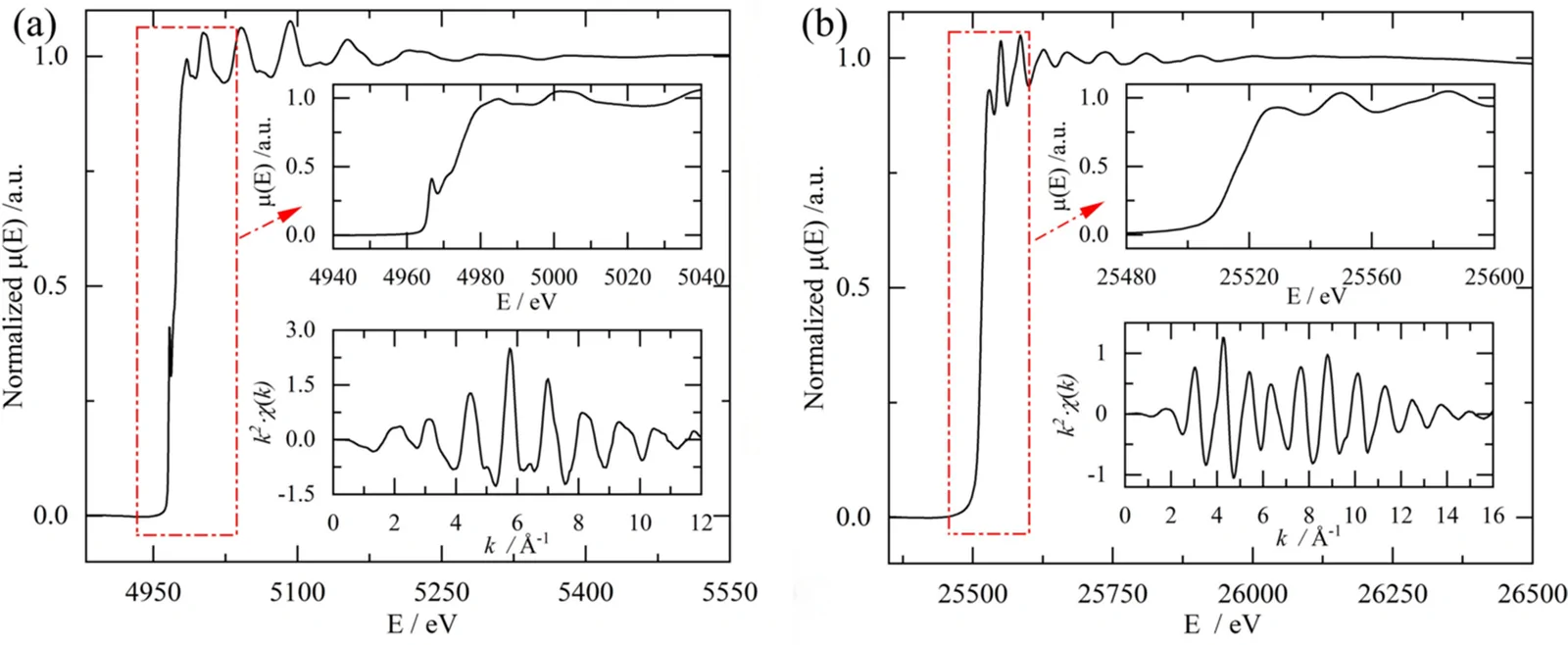
Normalized *K*-edge XAFS spectra of (*a*) Ti and (*b*) Ag acquired in conventional step-scan mode. The upper and lower insets in each panel display the zoomed-in XANES region and the *k*^2^-weighted EXAFS oscillations, respectively.

The accessible energy range of the monochromator is determined by the angular limits of the Bragg goniometer and the Si(111) channel-cut crystal optics. The monochromator can oscillate by up to ±2.1° around a selected central Bragg angle, corresponding to a maximum angular scan range of 4.2°. Because the angle-to-energy conversion is nonlinear, the energy window covered in a single oscillation depends on the selected central angle. To benchmark the practical operating energy range, conventional step-scan measurements were performed on standard Ti and Ag reference foils. The resulting high-quality *K*-edge spectra for both elements (Fig. 5 ▸) confirm an effective energy range of 5–26.5 keV for QXAFS measurements at BL11B.

#### 3.1.2. Transmission QXAFS

**Figure 6:**
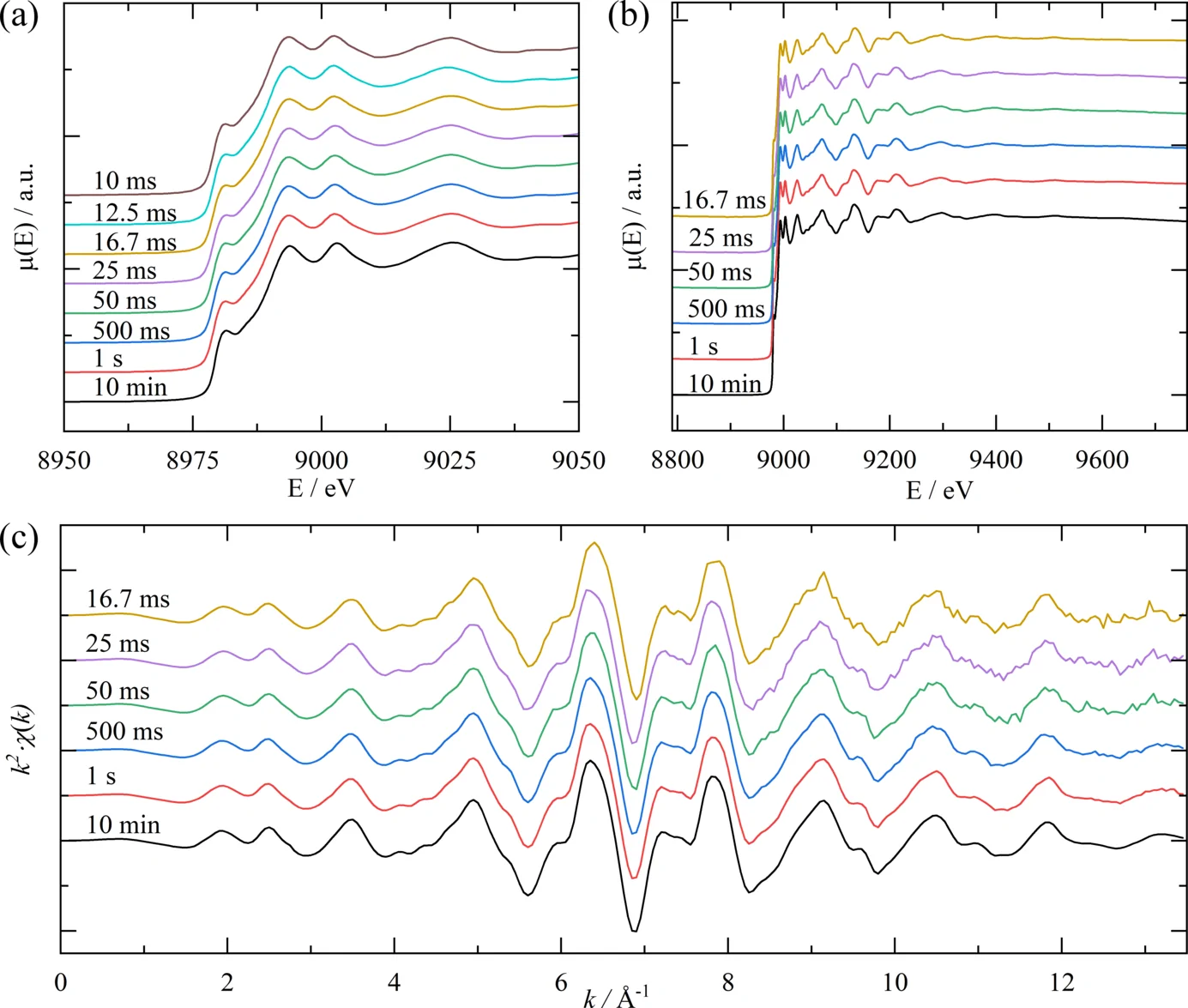
Normalized Cu *K*-edge (*a*) XANES and (*b*) full XAFS spectra of a Cu foil acquired with different scan times. (*c*) Comparison of the corresponding *k*^2^-weighted EXAFS oscillations. The scanning parameters for each oscillation frequency (single-scan acquisition time, total angular scan range, and energy scan range) are as follows: 0.5 Hz (1 s, 1.5°, ∼1110 eV); 1 Hz (500 ms, 1.38°, ∼1030 eV); 10 Hz (50 ms, 1.35°, ∼1010 eV); 20 Hz (25 ms, 1.36°, ∼1040 eV); 30 Hz (16.7 ms, 1.18°, ∼840 eV); 40 Hz (12.5 ms, 0.61°, ∼430 eV); 50 Hz (10 ms, 0.33°, ∼230 eV, XANES only). A conventional step-scan XAFS spectrum (10 min) is presented for comparison. All measurements were performed in transmission mode using nitro­gen-filled ionization chambers with an amplifier gain of 10^6^ V A^−1^.

To assess the temporal resolution and spectral quality of the platform, time-resolved Cu *K*-edge XANES and EXAFS spectra of a standard Cu foil were collected using atmospheric-pressure, nitro­gen-filled GICs. Fig. 6 ▸(*a*) presents the XANES spectra acquired at oscillation frequencies ranging from 0.5 to 50 Hz, together with a conventional step-scan spectrum for reference. The characteristic shoulder feature at 8981.3 eV remains discernible even at 50 Hz, corresponding to an acquisition time of 10 ms per spectrum, indicating that the main XANES features remain identifiable during rapid scanning. Furthermore, the EXAFS spectra [Figs. 6 ▸(*b*) and 6(*c*), and Fig. S10 of the supporting information] retain usable signal quality up to *k* ≃ 12.5 Å^−1^ at 30 Hz (16.7 ms per spectrum). This demonstrates the feasibility of tracking *in situ* oxidation state and local structure evolution on the millisecond timescale. However, the impact of hardware bandwidth on spectral fidelity under such ultrafast, wide-energy-range conditions requires more rigorous quantification, which is systematically addressed in Section 5.

#### 3.1.3. Fluorescence QXAFS of a dilute ZnO sample

**Figure 7:**
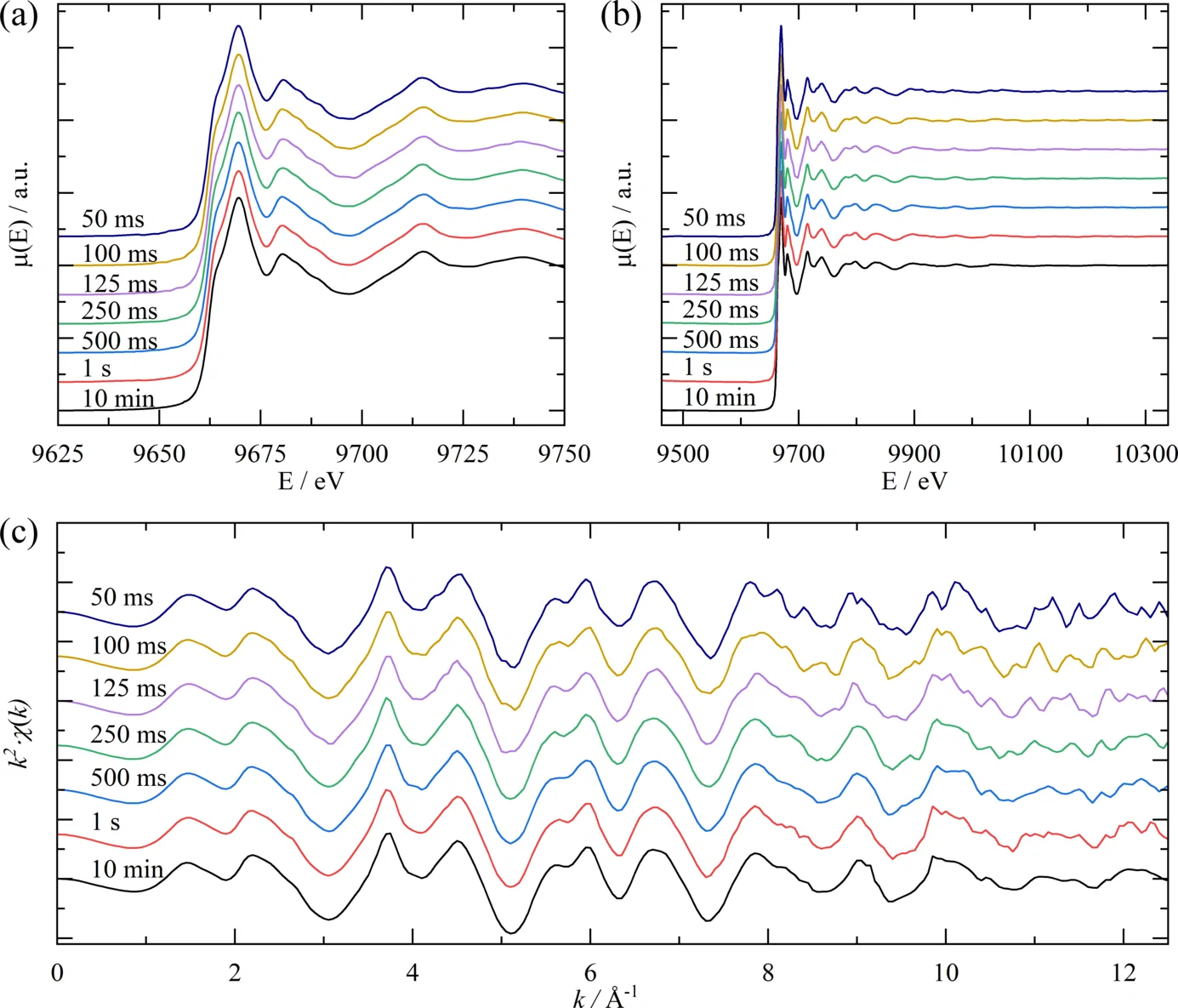
Normalized Zn *K*-edge (*a*) XANES and (*b*) full XAFS spectra of 1 wt% ZnO acquired with different scan times. (*c*) Comparison of the corresponding *k*^2^-weighted EXAFS oscillations. A conventional step-scan XAFS spectrum (10 min) is presented for comparison. All measurements were performed in fluorescence mode.

The performance of the fluorescence-mode QXAFS was evaluated by employing an atmospheric-pressure nitro­gen-filled GIC and a PIPS detector to monitor the incident *I*_0_ and fluorescence intensities *I*_f_, respectively. We measured a dilute 1 wt% ZnO sample at oscillation frequencies ranging from 1 to 10 Hz, as shown in Fig. 7 ▸. Although the signal-to-noise ratio of the EXAFS spectra in both *E*- and *k*-space understandably degrades as the oscillation frequency increases, the XANES region retains high spectral quality. This enables the practical tracking of valence changes and white-line shifts on timescales from 50 to 500 ms. For EXAFS, the noise level increases significantly at higher frequencies due to the inherently reduced photon statistics at shorter integration times. Nevertheless, an optimized acquisition time of 500 ms per spectrum (1 Hz oscillation) maintains discernible structural oscillations up to *k* ≃ 12.5 Å^−1^, providing signal quality sufficient for fitting structural parameters.

#### 3.1.4. Measurement of multiple edges

**Figure 8:**
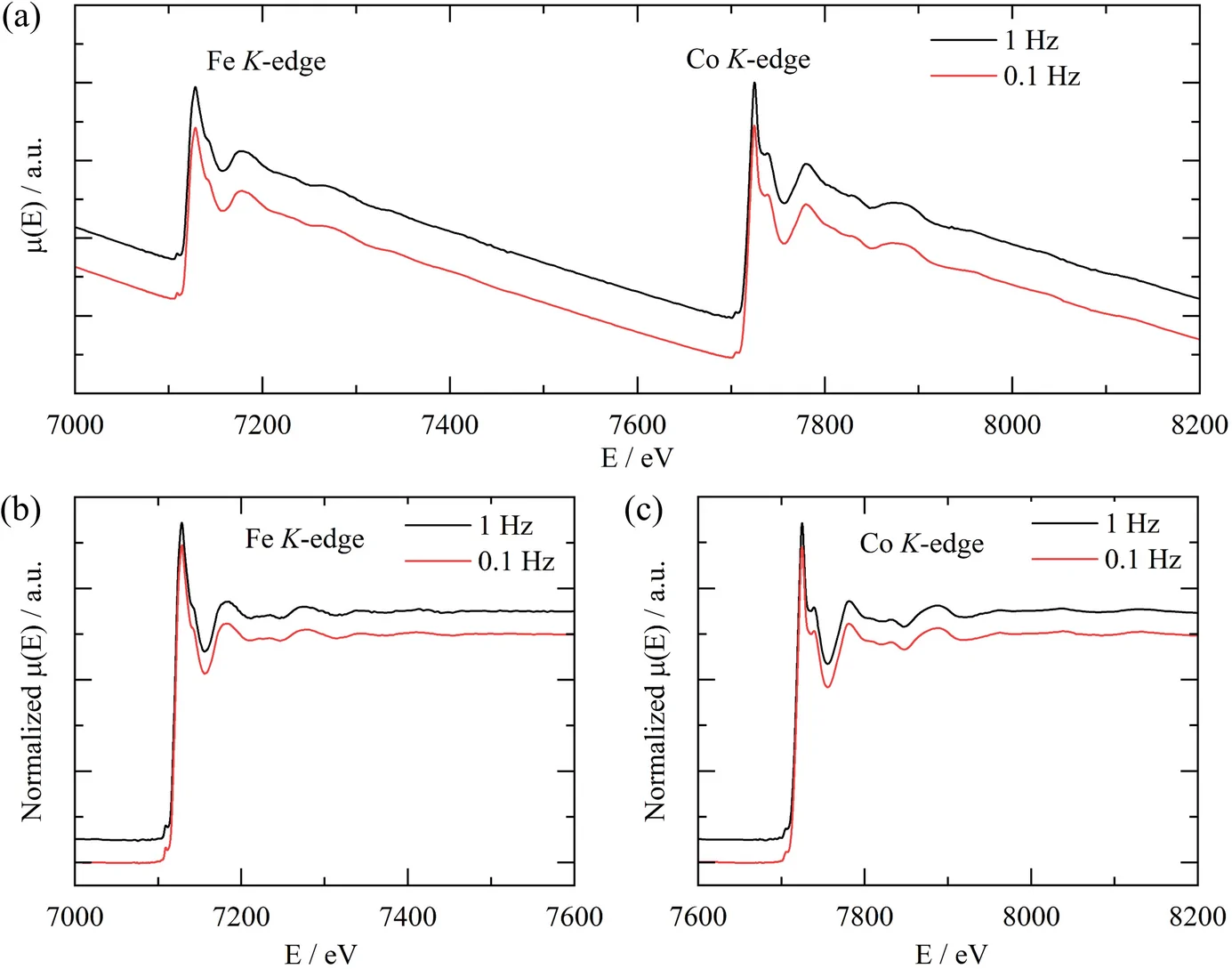
(*a*) Wide-range QXAFS spectra of Co_0.6_Fe_0.4_MoO_4_ covering both the Fe and Co *K*-edges in a single scan. Normalized *K*-edge XAFS spectra of (*b*) Fe and (*c*) Co extracted from the continuous scans. All measurements were performed at monochromator oscillation frequencies of 0.1 and 1 Hz. The spectra are vertically offset for clarity.

The large total angular scan range of the monochromator (up to 4.2°) provides a wide energy window within a single continuous scan. This makes it feasible to capture multiple closely spaced absorption edges within a single continuous scan, such as the *K*-edges of neighbouring 3*d* transition metals or the multiple *L*-edges of 5*d* elements. To demonstrate this, we collected the wide-range QXAFS spectra of a molybdate Co_0.6_Fe_0.4_MoO_4_ sample, capturing both the Fe and Co *K*-edges in a single sweep at oscillation frequencies of 0.1 and 1 Hz. As shown in Fig. 8 ▸(*a*), the raw full-range spectra retain identifiable features across both absorption edges. Furthermore, the normalized absorption spectra for the Fe [Fig. 8 ▸(*b*)] and Co [Fig. 8 ▸(*c*)] *K*-edges collected at 0.1 Hz show similar edge positions and spectral profiles to those acquired at 1 Hz. Such concurrent multi-element monitoring may be advantageous for investigating complex, dynamically evolving systems such as multimetallic catalysts and battery materials.

### 3.2. Scan-direction- and frequency-dependent spectral deviations

**Figure 9:**
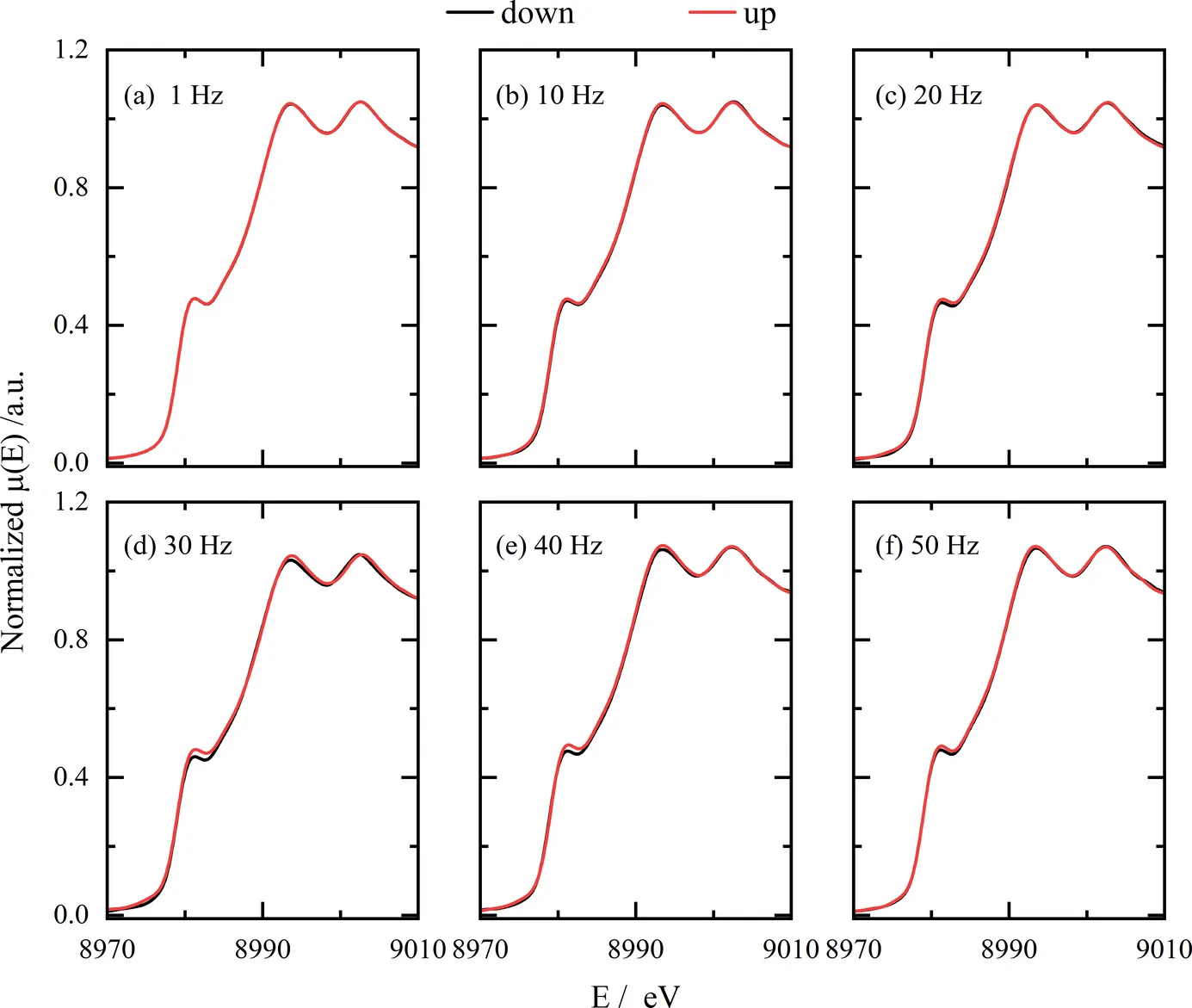
Comparison of normalized Cu *K*-edge XANES spectra acquired in the decreasing-angle (down) and increasing-angle (up) directions at oscillation frequencies of (*a*) 1, (*b*) 10, (*c*) 20, (*d*) 30, (*e*) 40 and (*f*) 50 Hz, corresponding to acquisition times of 500, 50, 25, 16.7, 12.5 and 10 ms per spectrum, respectively. Spectra from both directions were processed using the same angular-binning, filtering and fixed energy-calibration procedure. No spectrum-by-spectrum energy realignment was applied.

To assess the consistency of rapid-scan measurements, Cu *K*-edge XANES spectra acquired during the decreasing-angle (down) and increasing-angle (up) branches were compared over the investigated oscillation-frequency range (Fig. 9 ▸). The principal absorption edge, shoulder and post-edge features are reproduced in both directions, although small differences remain in the apparent edge position and local spectral shape. These differences vary non-monotonically with oscillation frequency.

Detailed edge-position statistics and encoder-based kinematic analyses are provided in Section S7 of the supporting information. These analyses show that the angular range and local scan rate also vary with operating conditions. The residual differences may therefore reflect the combined effects of the actual monochromator trajectory and the finite response of the measurement chain, rather than a simple frequency-proportional mechanical lag.

## 4. Application: *in situ* tracking of electrochemical reconstruction

**Figure 10:**
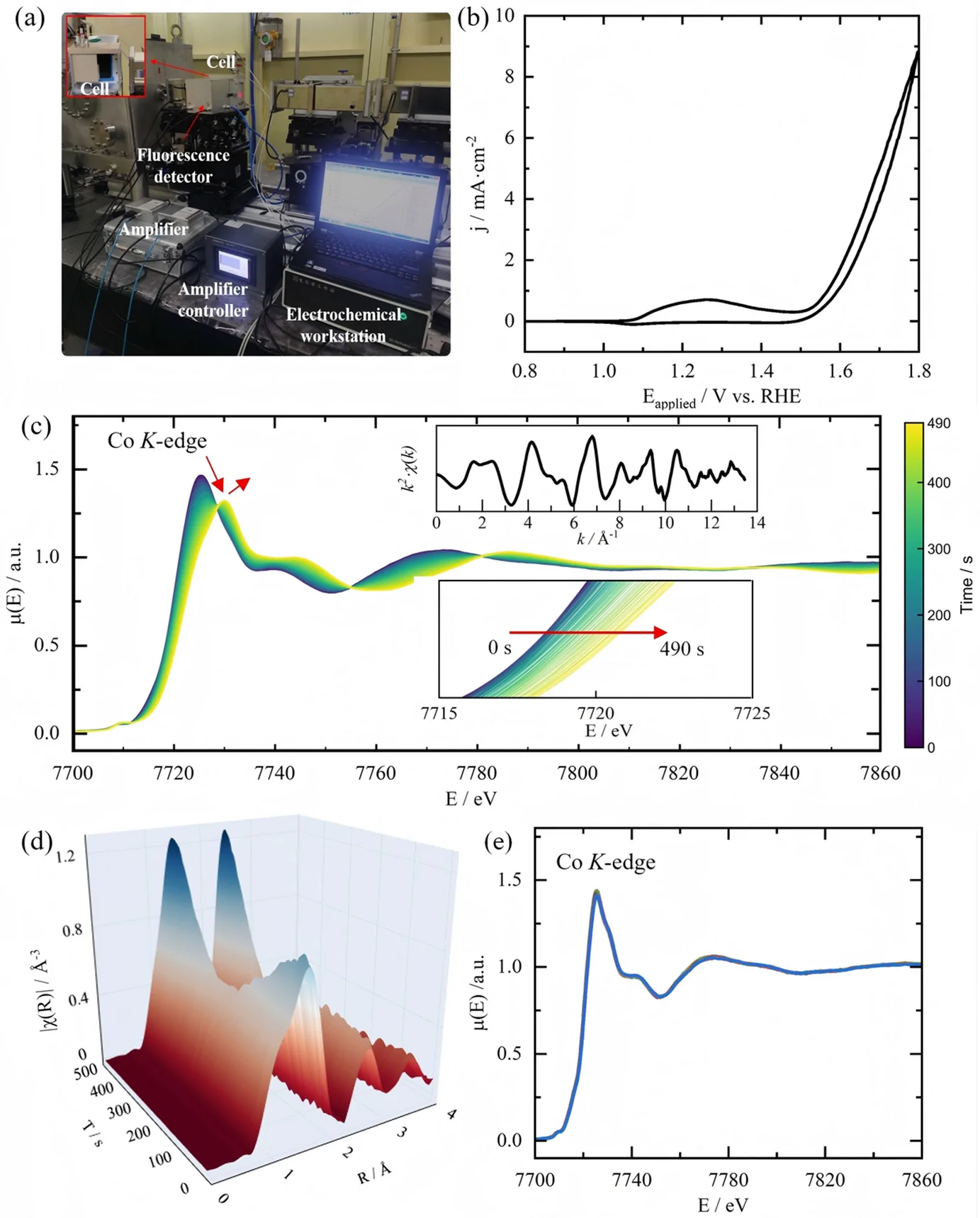
*In situ* QXAFS characterization of β-CoMoO_4_ under OER conditions. (*a*) Photograph of the *operando* electrochemical QXAFS setup at the BL11B beamline. (*b*) First-cycle CV curve of β-CoMoO_4_ in 1 *M* KOH (pH = 13.5) with a potential window of 0.8–1.8 V versus RHE at a scan rate of 2.5 mV s^−1^. (*c*) *Operando* time-resolved Co *K*-edge QXAFS spectra collected during the CV scanning. The upper and lower insets provide a magnified view of the *k*^2^-weighted EXAFS and the absorption-edge (XANES) regions, respectively. (*d*) 3D surface plot of the Fourier-transformed EXAFS spectra in *R*-space, illustrating the dynamic evolution of Co coordination structures as a function of the applied potential. (*e*) Selected XANES spectra extracted every 100 s (5 s per spectrum, 1000 spectra in total) under the pH = 12 condition. The spectra show no systematic edge shift or line-shape change within the observed variation, indicating no significant structural reconstruction within 5000 s.

To demonstrate the practical utility of the platform, we performed an *in situ* QXAFS study on the electrochemical reconstruction of β-CoMoO_4_, a pre-catalyst for the oxygen evolution reaction (OER) (Zhong *et al.*, 2025 ▸). The experimental setup is shown in Fig. 10 ▸(*a*). QXAFS spectra during cyclic voltammetry (CV) in 1 *M* KOH were recorded using an in-house-developed fluorescence detection system based on the original Lytle detector design (Lytle *et al.*, 1984 ▸). The catalyst was deposited on carbon paper as the working electrode, while Hg/HgO and a graphite rod were used as the reference and counter electrodes, respectively. CV scanning between 0.8 and 1.8 V versus the reversible hydrogen electrode (RHE) was recorded at a scan rate of 2.5 mV s^−1^, and the Co *K*-edge QXAFS spectra were simultaneously collected with an oscillation frequency of 0.1 Hz (5 s per spectrum). This acquisition condition was chosen to match the comparatively slow catalyst reconstruction observed on the several-hundred-second timescale. At the applied scan rate, the potential changed by only ∼12.5 mV during each 5 s spectrum, which was sufficient for tracking the *operando* evolution in the present case. Higher-frequency acquisition followed by temporal averaging is also a viable alternative for faster or initially unknown kinetics.

The first-cycle CV curve at pH = 13.5 is shown in Fig. 10 ▸(*b*). The time-resolved XANES spectra in Fig. 10 ▸(*c*) reveal a gradual positive shift of the absorption edge and a decrease in the white-line intensity with increasing applied potential, indicating the oxidation of Co ions and reconstruction of the catalyst. More importantly, distinct isosbestic points are observed in the QXAFS spectra, consistent with an interconversion between two dominant spectral species during the reconstruction process. After approximately 490 s, the spectral shape remains unchanged, suggesting that no further detectable spectral evolution occurred under the present measurement conditions. Fourier-transform analysis of the EXAFS spectra in Fig. 10 ▸(*d*) reveals that the Co coordination environment undergoes continuous evolution over the first 490 s, further supporting the rapid reconstruction of the catalyst. The high signal-to-noise ratio of the EXAFS data presented in the inset of Fig. 10 ▸(*c*) demonstrates the suitability of the QXAFS platform for dynamic structural studies.

For comparison, QXAFS experiments conducted at pH = 12 exhibit negligible variations in the XANES spectra over a 5000 s period [Fig. 10 ▸(*e*)]. Compared with pH = 13.5, the milder alkaline condition at pH = 12 showed substantially less spectral evolution within the investigated time window, suggesting that the reconstruction is promoted at higher alkalinity under the present experimental conditions. The spectral consistency also demonstrates long-term stability of the QXAFS platform during *in situ* measurements.

This *in situ* demonstration confirms the platform’s utility for tracking *operando* structural evolution during electrocatalysis under realistic experimental conditions. Since the 0.1 Hz acquisition used here represents a relatively conservative operating regime, the following section systematically examines the frequency-dependent noise behaviour and practical performance limits of the QXAFS platform.

## 5. Physical limits and methodological guidelines for QXAFS

### 5.1. Methodology of residual-based noise quantification

The spectral quality and performance limits of the QXAFS platform were assessed using the pre-edge residual noise magnitude (σ), defined as the root-mean-square (RMS) magnitude of the residuals within the pre-edge region. This region was selected because its near-zero slope (dμ/d*E* ≃ 0) reduces sensitivity to small energy-axis variations, while the absence of pronounced absorption features and EXAFS oscillations minimizes contributions from actual spectral structure. To ensure statistical consistency across all analysed oscillation frequencies, the oversampled raw data were rebinned onto a uniform angular grid (*e.g.* 0.2 arcsec per bin), establishing a consistent coordinate spacing regardless of the scan rate. Only down-scan spectra were included in the analysis. For each valid angular bin, the mean absorption coefficient of all contributing spectra was used as a bin-wise reference, and the residual of each spectrum was calculated relative to this reference. The residual noise magnitude (σ) for a specific spectral region was quantified by the following equation, with the detailed procedure described in Section S8 of the supporting information,
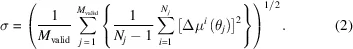
Here, *M*_valid_ is the total number of angular bins satisfying*N*_*j*_/*N*_total_ ≥ 70%, *N*_*j*_ is the number of spectra contributing valid data to the *j*th angular bin, and *N*_total_ is the total number of consecutive spectra included in the analysis. For a valid bin centred at θ_*j*_, 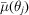 denotes the mean absorption coefficient of the *N*_*j*_ contributing spectra, and 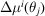 = 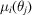 − 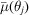 is the residual of the *i*th spectrum from this local bin-averaged reference.

### 5.2. Physical origins of noise scaling and instrumental limits

**Figure 11:**
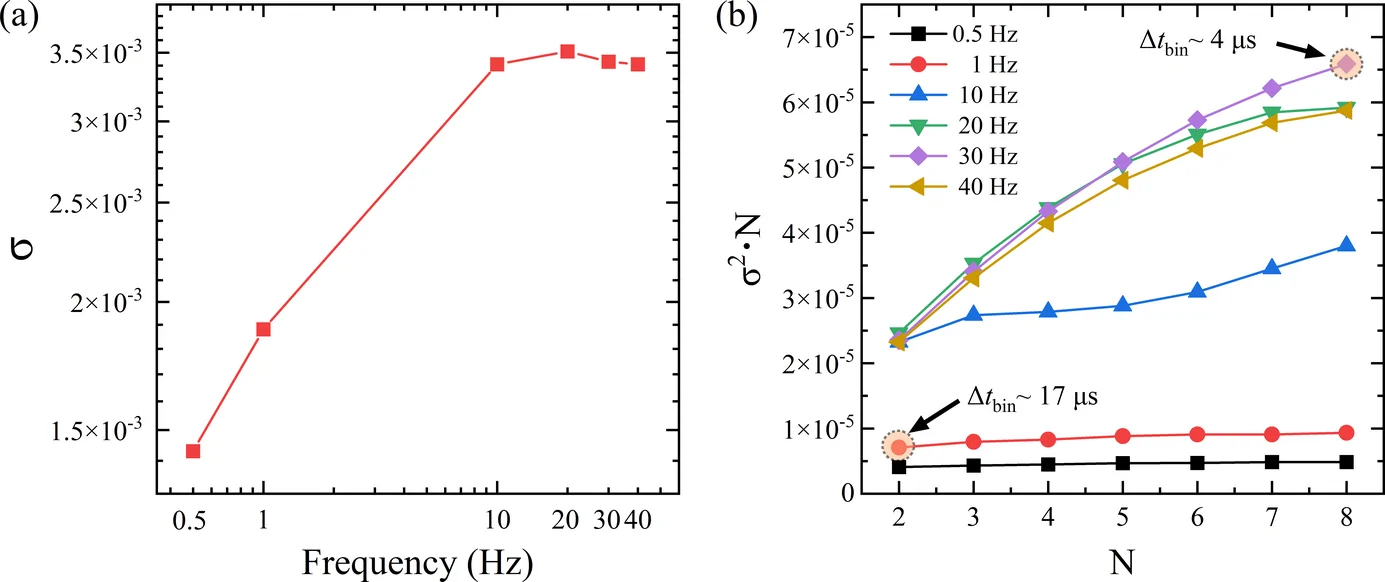
Quantitative noise evaluation and statistical independence analysis of the QXAFS platform. (*a*) Pre-edge residual noise (σ) as a function of oscillation frequency. (*b*) Scaled variance (σ^2^*N*) versus bin-merging factor (*N*). The upward deviation from independent statistical behaviour at high frequencies (≥10 Hz) quantitatively indicates increasing correlation between neighbouring data points. Annotated points (Δ*t*_bin_) contrast the near-statistical regime at low frequency (1 Hz) with increased covariance at high frequency (30 Hz), which is mainly associated with the finite temporal response of the detector–amplifier–readout chain: when the effective bin interval becomes too short, signals from adjacent bins partially overlap and lose statistical independence.

To understand the operational limits of the QXAFS platform, we evaluated the noise scaling behaviour from kinematic and statistical perspectives. As shown in Fig. 11 ▸(*a*), the residual noise σ generally increases from 0.5 to 10 Hz, although the varying slope on the log–log scale indicates non-uniform noise scaling. Above 10 Hz, the departure from statistical scaling becomes more pronounced. This behaviour is consistent with finite-response effects becoming increasingly important relative to shot noise, with the detection chain acting as a low-pass filter at short bin-transit times.

To further examine this interpretation, we evaluated the statistical independence of the data, which is fundamentally governed by the relationship between the dwell time per bin (Δ*t*_bin_, the physical transit time) and the specific response time of our detection system (10%–90% rise time *t*_r_ ≃ 20 µs). This temporal dependency is explicitly demonstrated in Fig. 11 ▸(*b*) using the scaled variance (σ^2^*N*), where *N* represents the number of digitized angular increments (0.1 arcsec each) grouped within each bin. In a purely shot-noise-limited system, this value remains constant. At 1 Hz (*N* = 2), the transit time is Δ*t*_bin_ ≃ 17 µs. Since Δ*t*_bin_ is comparable with the system rise time (1*t*_r_), the data retain sufficient statistical independence to form a nearly horizontal plateau. In contrast, at 30 Hz (*N* = 8), where Δ*t*_bin_ (≃ 4 µs) is significantly shorter than *t*_r_, the finite response of the detection chain introduces increasing correlation between adjacent bins. Because the detector–amplifier–readout system cannot fully respond within such a short interval, part of the signal recorded in one bin carries over into the next bin. This temporal overlap breaks the assumption of independent events, causing the sharp upward deviation observed in the scaled variance. Under these conditions, reducing the bin size does not necessarily provide additional independent spectral information. The stark contrast between the threshold condition (∼ 1*t*_r_) and the highly coupled state (≪1*t*_r_) thus reveals a critical pitfall in data processing: since these highly coupled bins are merely artificially over-segmenting a finite-response-broadened signal, attempting to resolve finer features using a bin size whose transit time (Δ*t*_bin_) is shorter than the system’s response time merely creates a ‘false resolution’. To accurately reflect the system’s true resolving power and to establish a statistically independent baseline, the data binning interval should correspond to a physical transit time of at least 2*t*_r_.

### 5.3. Response-based estimates for data reduction and scan design

Valid QXAFS experimental design requires balancing two fundamental, often conflicting, constraints. To resolve actual spectral features according to the Nyquist theorem, the energy bin size must be sufficiently small (*e.g.*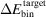 ≤ 0.5 eV, approximately one-third of the core-hole lifetime broadening for the Cu *K*-edge). However, to prevent finite-response-related covariance between adjacent bins, the bin size must physically exceed the energy interval swept by the monochromator during twice the system’s rise time (2*t*_r_). During a continuous sweep, the maximum instantaneous velocity (*v*_max_) dictates the minimum valid bin size: Δ*E*_min_ = *v*_max_2*t*_r_. Assuming a nominal sinusoidal scan trajectory, *v*_max_ peaks near the centre of the oscillation range, yielding *v*_max_ ≃ π*fE*_range_. Therefore, to ensure both statistical reliability and spectral fidelity, the experimental parameters must satisfy a dual constraint,
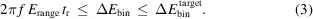
This inequality provides a practical criterion for optimizing scan parameters (*f* and *E*_range_) according to the specific hardware limits of a given beamline. Taking our detection system (*t*_r_ ≃ 20 µs) as a benchmark, this constraint establishes the following operational boundaries:

(i) Conventional kinetics (full EXAFS). For reactions permitting slower scans, resolving a 0.5 eV target over a full 1000 eV EXAFS range limits the maximum oscillation frequency of the monochromator to *f* = 4 Hz (125 ms per spectrum). Operating within this boundary ensures high-fidelity EXAFS spectra with reduced finite-response-induced spectral distortion.

(ii) Ultrafast kinetics (the 30 Hz trade-off). Attempting a full 1000 eV scan at 30 Hz inflates the minimum valid bin size to ∼3.8 eV, fundamentally violating the ≤0.5 eV requirement. To capture ultrafast dynamics at 30 Hz while rigorously maintaining the target energy-binning interval of 0.5 eV, researchers must drastically compress the scanning range. The constraint equation dictates a maximum scan range of *E*_range_ ≤ 130 eV, restricting the measurement to the XANES region.

Accordingly, under the present response and sampling conditions, 30 Hz acquisition is better suited to narrow-range XANES than to full-range EXAFS.

### 5.4. Current limitations and future upgrade pathways

While the platform meets the demands of most *in situ* experiments, the quantitative evaluation performed in this study highlights clear pathways for further optimization:

(i) Detection system upgrades for dilute systems. For fluorescence-mode QXAFS on dilute samples, the temporal resolution is fundamentally constrained by the statistical flux from the bending magnet and the limited solid-angle coverage of the single PIPS detector. Adopting a multi-element PIPS array would significantly enhance the geometric collection efficiency. Such a configuration optimally balances high photon throughput with the high-frequency dynamic response required by the QXAFS framework, driving simultaneous improvements in both signal-to-noise ratio and effective time resolution.

(ii) Advanced data-driven workflows. Machine-learning denoising and chemometric methods do not remove the fundamental information loss associated with detector-response, bandwidth or sampling limitations. Nevertheless, when sufficient spectral signal is retained, they may still serve as useful complementary tools for QXAFS analysis. Advanced denoising methods (Rüscher *et al.*, 2022 ▸; Du *et al.*, 2025 ▸) may help reduce predominantly uncorrelated noise, and chemometric approaches such as multivariate curve resolution-alternating least squares (MCR-ALS) (Garetto *et al.*, 2025 ▸) may aid the statistical interpretation of evolving or partially overlapping spectral components. In this way, data driven workflows may improve the practical usability of the high-*k* region of sub-20 ms EXAFS data and support more robust analysis near the instrumental limits identified in this work.

## 6. Conclusion

A millisecond-to-second QXAFS platform has been successfully established at SSRF BL11B for time-resolved X-ray absorption studies under *in situ* and *operando* conditions. By integrating a torque-motor-driven monochromator with synchronized high-speed detection and automated data reduction, the platform enables rapid measurements over 5–26.5 keV, including 10 ms XANES, 16.7 ms full-range EXAFS and 50 ms fluorescence-mode XANES for dilute samples.

A key outcome of this work is the quantitative definition of practically useful scan conditions for the present instrument. The results show that the experimentally meaningful time resolution is governed not only by the nominal scan speed but also by the combined effects of scan range and the finite response of the detection and readout system. For the current BL11B configuration, this leads to recommended monochromator oscillation frequencies of about 4 Hz for full-range EXAFS scans spanning roughly 1000 eV, whereas frequencies around 30 Hz are appropriate when the scan window is reduced to about 130 eV in XANES mode. These recommendations are therefore set by the response performance of the present detection chain and provide practical guidance for experiment design on this platform.

Together with the *in situ* electrocatalysis demonstration, these results establish BL11B as a user-oriented QXAFS capability and provide a quantitative basis for balancing temporal resolution, spectral coverage and data fidelity in future rapid-scan XAFS experiments.

## Supplementary Material

Sections S1 to S8 inlcuding Tables S1 to S5 and Figures S1 to S14. DOI: 10.1107/S1600577526009781/ret5005sup1.pdf

## Figures and Tables

**Table 1:** Comparison of different QXAFS implementation strategies

QXAFS type	Oscillatory drive	Monochromator crystal	Time scale	First implemented beamlines
Continuous scan	Stepper motor	Double-crystal	Seconds	RÖMO I/II (HASYLAB)
Piezo-driven	Piezo actuators	Double-crystal	∼5 ms (XANES only)	BW1 (HASYLAB)
Eccentric cam-driven	Eccentric disk/shaft	Channel-cut	10–100 ms	ROCK (SOLEIL)
Direct-driven	Gearless servo motor	Channel-cut	10–100 ms	BL33XU (SPring-8), SuperXAS (SLS)

**Table 2:** Specifications of the BL11B beamline

Parameter	Specification
Storage-ring energy	3.5 GeV
Current	300 mA
Magnetic field	1.27 T
Critical energy	10.346 keV
Beamline energy range	5–30 keV
QXAFS operating range	5–26.5 keV
Energy resolution (Δ*E*/*E*)	1.5 × 10^−4^ at 10 keV, Si (111)
Flux at sample	5 × 10^11^ photons s^−1^ at 10 keV
Spot size	200 µm (H) × 250 µm (V)

## Data Availability

The *QXAFS Data Studio* software developed in this work is currently maintained as an in-house tool specifically optimized for the BL11B beamline at SSRF. The executable software and related documentation are available from the corresponding authors upon reasonable request. The raw experimental data that support the findings of this study are also available from the corresponding authors upon reasonable request.
