# Supplementary material for: Construction and performance of the millisecond-to-second QXAFS platform at SSRF BL11B for in situ dynamic characterization
Source: J Synchrotron Radiat. 2026 Oct 29;33(Pt 6):1834–44. doi: 10.1107/S1600577526009781 (PMC13753870; doi:10.1107/S1600577526009781)
Supplement: Supplementary file 1 [file s-33-01834-sup1.pdf]

JOURNAL OF SYNCHROTRON RADIATION

Volume 33 (2026)

Supporting information for article:

Construction and performance of the millisecond-to-second QXAFS platform at SSRF BL11B for *in situ* dynamic characterization

Yu Chen, Yu Li, Qian Gao, Tao Gan, Shuo Zhang and Jiong Li

S1. Experimental evaluation of the Si(111) crystal performance at the Ag *K*-edge

Table S1 lists the *K*-edge energies and intrinsic core-hole lifetime broadenings of representative 4d elements accessible at BL11B for reference. To assess whether the Si(111) channel-cut crystal provides sufficient spectral performance near the upper end of the routinely accessible energy range, Ag *K*-edge spectra of the same Ag foil were compared with spectra measured using the Si(311) monochromator (Fig. S1).

As shown in Fig. S1, the Ag *K*-edge spectra collected with Si(111) and Si(311) exhibit essentially identical edge positions and reproduce the same principal XANES and EXAFS features. The main differences are limited to moderate broadening of subtle near-edge features and some damping of EXAFS amplitude in the Si(111) data. The corresponding FT-EXAFS spectra also show consistent peak positions and similar overall profiles for the principal and higher-shell contributions, indicating that the local coordination-shell information is preserved. The reduced EXAFS amplitude in the Si(111) data is attributed mainly to instrument-configuration-dependent resolution effects and does not alter the main coordination-shell assignments.

These results show that, although Si(111) provides lower intrinsic energy resolution than Si(311), it remains sufficient for the spectral analysis required in the present work, including edge-position tracking and local coordination analysis.

Table S1 *K*-edge energies and core-hole lifetime broadenings of selected 4d elements.

Element	*K*-edge (eV)	Core-hole lifetime broadening (eV)
Nb	18986	4.14
Mo	20000	4.52
Ru	22117	5.33
Rh	23220	5.77
Pd	24350	6.24
Ag	25514	6.75

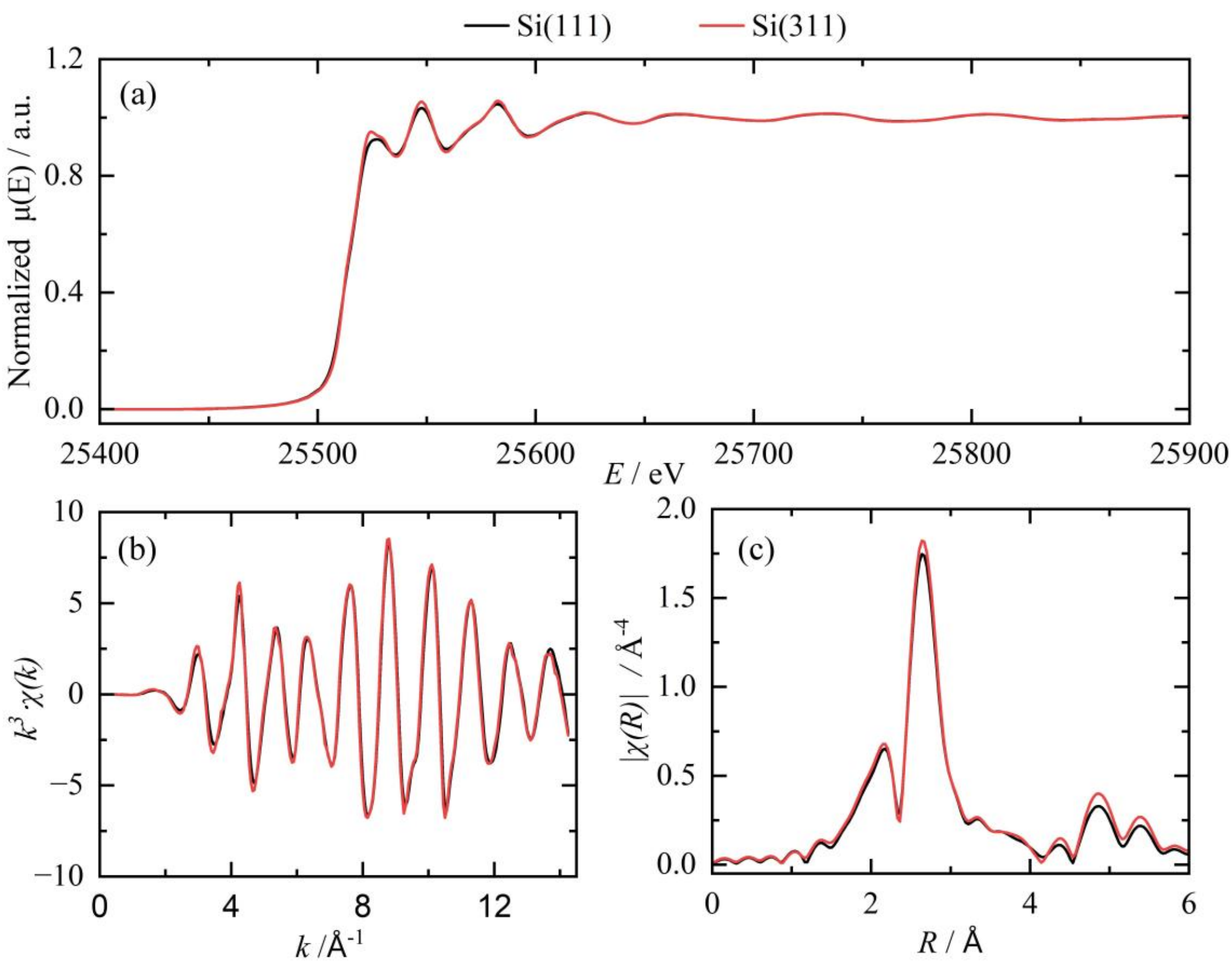

Figure S1 Comparison of the Ag *K*-edge XAFS spectra of the same Ag foil measured using Si(111) CCM and Si(311) DCM. (a) normalized XAFS spectra. (b) k^3-weighted EXAFS oscillations. (c) Fourier-transform magnitudes in *R*-space. The black and red curves represent the spectra measured using Si(111) CCM and Si(311) DCM, respectively.

S2. Automatic gas-mixing system for the ionization chambers

BL11B is equipped with an automatic gas-mixing system for ionization chambers, as shown in Fig. S2. The system is supplied with high-purity He, N_2, Ar and Kr, with each gas line regulated independently by a mass-flow controller. After the target element and absorption edge are selected in the control interface, the required gas composition is calculated according to the X-ray energy and the active length of the ionization chamber. An absorption of approximately 10% is used as the initial setting to provide sufficient ionization current without introducing excessive attenuation of the incident beam.

The calculated gas composition is converted into individual flow-rate setpoints. Under steady-flow conditions, the volume fraction of gas i is determined by

$$x_i = \frac{Q_i}{\sum_j Q_j},$$

where Q_i is the flow rate of the corresponding gas line. The control interface displays the selected gases, calculated fractions, flow-rate setpoints and measured flow rates, and allows the gas mixture to be directed to any of the four ionization chambers. The mixed gas is continuously supplied to the selected chamber during operation.

For the benchmark measurements reported in this work, flowing N_2 at approximately atmospheric pressure was used throughout to ensure that the detector conditions remained unchanged when comparing different oscillation frequencies. Although higher gas pressure could in principle increase the absorption efficiency, the ionization signals from the standard foils were sufficient under the present near-atmospheric-pressure conditions. Therefore, during routine flow-through operation, the ionization-chamber absorption efficiency is adjusted primarily by changing the gas composition rather than by increasing the total pressure. The automatic mixing capability is routinely used in user experiments to adapt the detector gas to the absorption-edge of the measured element.

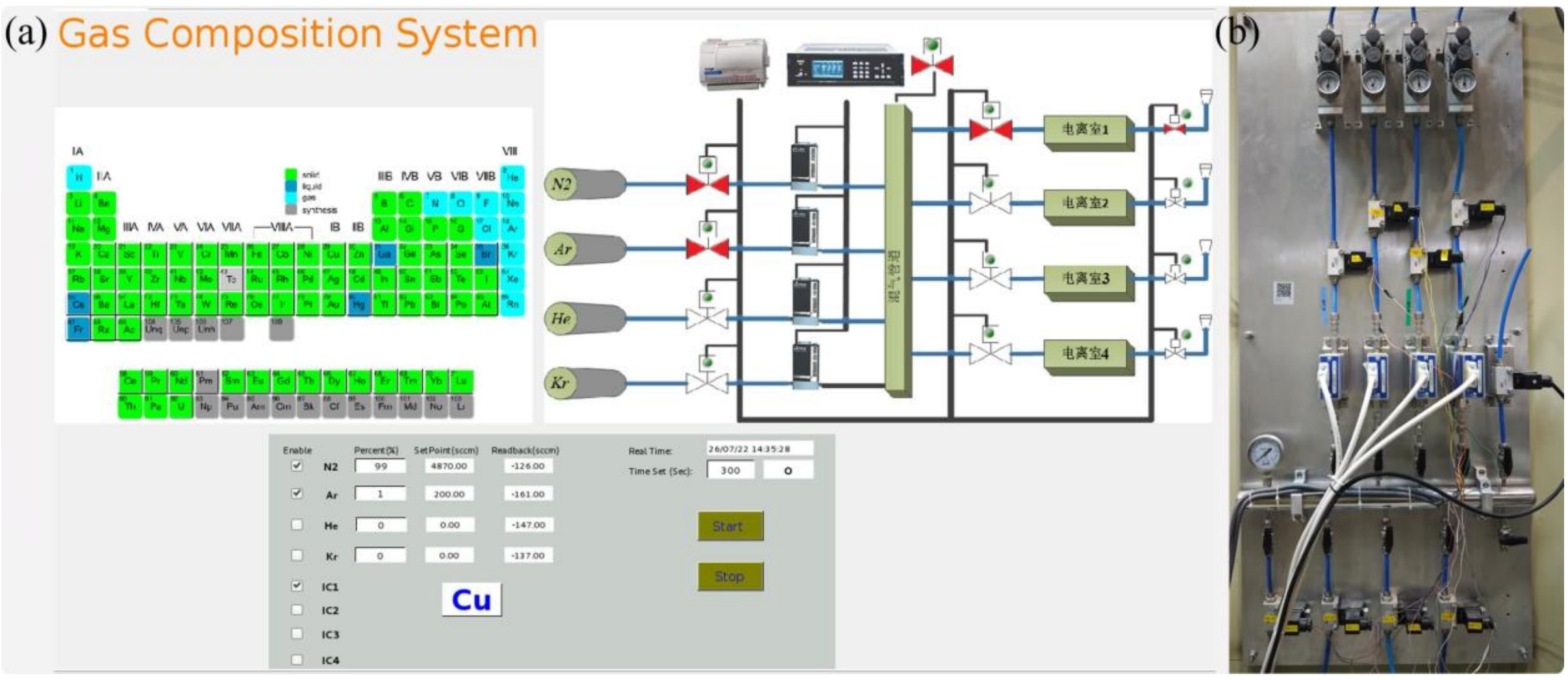

Figure S2 Automatic gas-mixing system for the ionization chambers at BL11B. (*a*) Control interface used to select the target element, gas species, mixture fractions, flow-rate setpoints and ionization chamber. The displayed Cu configuration is an example of the operating interface and does not represent the N_2 condition used for all benchmark measurements. (*b*) Photograph of the gas-mixing panel, including the He, N_2, Ar and Kr gas lines, flow-control units, mixing manifold and distribution lines to the four ionization chambers.

S3. Detection systems for QXAFS

S3.1. Fast-Lytle ionization chamber for low-speed *operando* measurements

For fluorescence measurements in the lower-speed regime, including the 0.1 Hz *operando* experiment reported in this work, we used a custom multi-grid ionization chamber (fast-Lytle), inspired by the pioneering designs (Lytle *et al*., 1984; Suzuki *et al*., 2002). The detector was engineered to enhance ion collection efficiency and minimize electron drift times. The temporal response was evaluated using a mechanical X-ray chopper. As shown in Fig. S3, the measured 10%–90% rise time of the fast-Lytle detection system was approximately 650 µs under a 1000 V bias. This response time was sufficient for the sub-second measurements performed during the initial commissioning and for the 0.1 Hz *operando* experiment reported in this work.

However, for dilute samples and for fluorescence QXAFS in the tens-of-milliseconds regime, the response characteristics of the fast-Lytle detector were not sufficient under the conditions used here. The fluorescence measurements in that regime therefore employed the PIPS-based detection system described in Section S3.2.

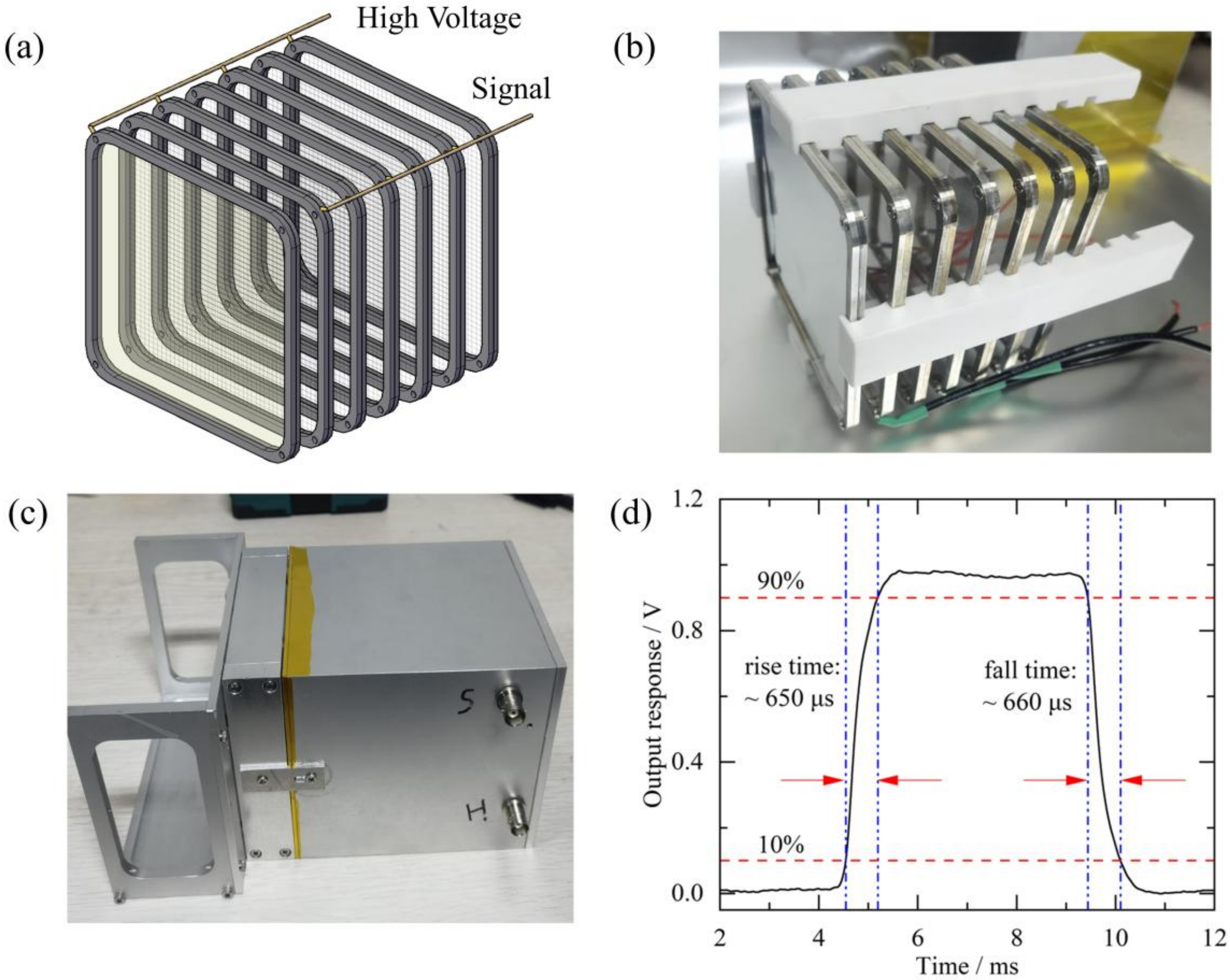

Figure S3 Design and performance of the multi-grid ionization chamber (fast-Lytle). (*a*) 3D schematic model of the detector. (*b*) Photograph of the internal multi-grid assembly. (*c*) Photograph of the fully assembled detector integrated with the sample chamber and slits. (*d*) Typical output voltage

waveforms demonstrating the temporal response of the fast-Lytle detection system, with an indicated 10%–90% rise time of ~650 µs.

S3.2. PIPS detector optimization for millisecond fluorescence QXAFS

For fluorescence-mode QXAFS on the millisecond timescale, balancing the system response time with signal integrity is critical. The 50-mm-diameter PIPS detector exhibits a significant junction capacitance, which can induce severe signal ringing in high-bandwidth current amplifiers, thereby distorting sharp absorption edges and fine structural features. To mitigate this, a feedback capacitor was integrated into the preamplifier circuit to function as a low-pass filter.

As illustrated in Fig. S4, we systematically evaluated the detector's response to pulsed fluorescence signals across a range of filter time constants (1 to 300 µs). While longer time constants (e.g., 100 or 300 µs) effectively eliminate ringing, they significantly prolong the rise and fall times, which would compromise the required millisecond-scale temporal resolution. Conversely, while shorter constants (1–30 µs) leave visible residual oscillations, they preserve the sharp temporal response required for fast scanning. Based on the measured waveforms, a time constant of 30 µs was selected for the measurements reported here as a compromise between residual ringing and rise/fall time.

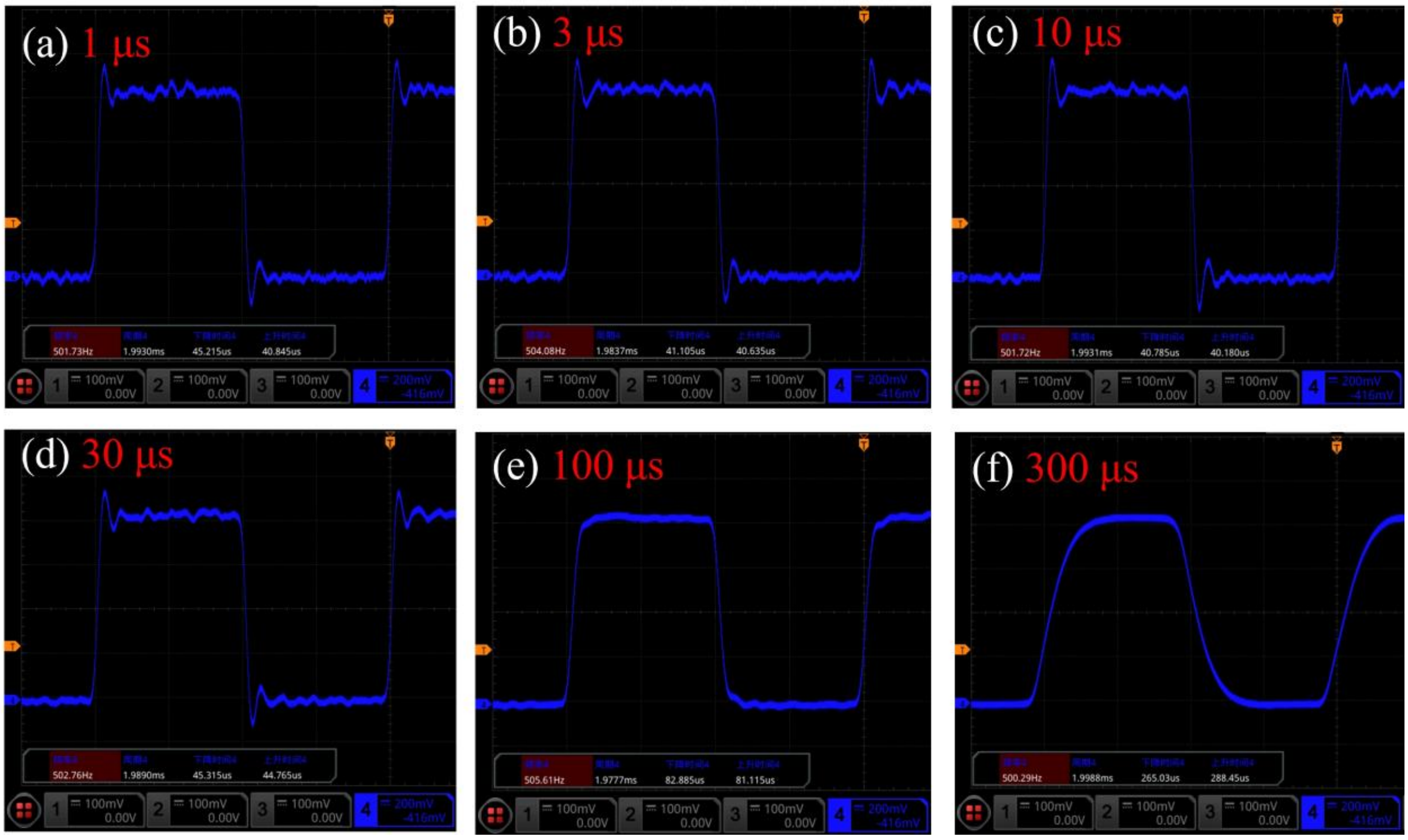

Figure S4 Oscilloscope traces of the PIPS detector response to pulsed fluorescence signals under different filter time constants implemented via feedback capacitance in the preamplifier: (*a*) 1 µs, (*b*) 3 µs, (*c*) 10 µs, (*d*) 30 µs, (*e*) 100 µs, and (*f*) 300 µs. Longer time constants effectively suppress signal ringing but increase the overall system response time.

S3.3. Detector linearity and summary

The linear response of the integrating fluorescence detectors was evaluated using a fixed-geometry scattering measurement. The incident intensity was monitored by an ionization chamber, and the scattered signal was recorded simultaneously by the detector under evaluation. A homogeneous PTFE block was used to provide a stable scattering signal while reducing the likelihood of detector saturation and local heating compared with a strongly absorbing metal sample.

As shown in Fig. S5, the detector outputs varied linearly with the incident-flux monitor over the investigated ranges. At 9 keV, the fast-Lytle detector gave $R^2 = 0.9996$ up to an incident flux of 2.7×10^{11} photons s^{-1} [Fig. S5(*a*)]. The PIPS detector gave $R^2 > 0.9999$ at 10, 15, and 20 keV, including measurements up to 5.5×10^{11} photons s^{-1} at 10 keV [Fig. S5(*b*)]. These results indicate that both detectors operated within their linear response ranges under the tested conditions.

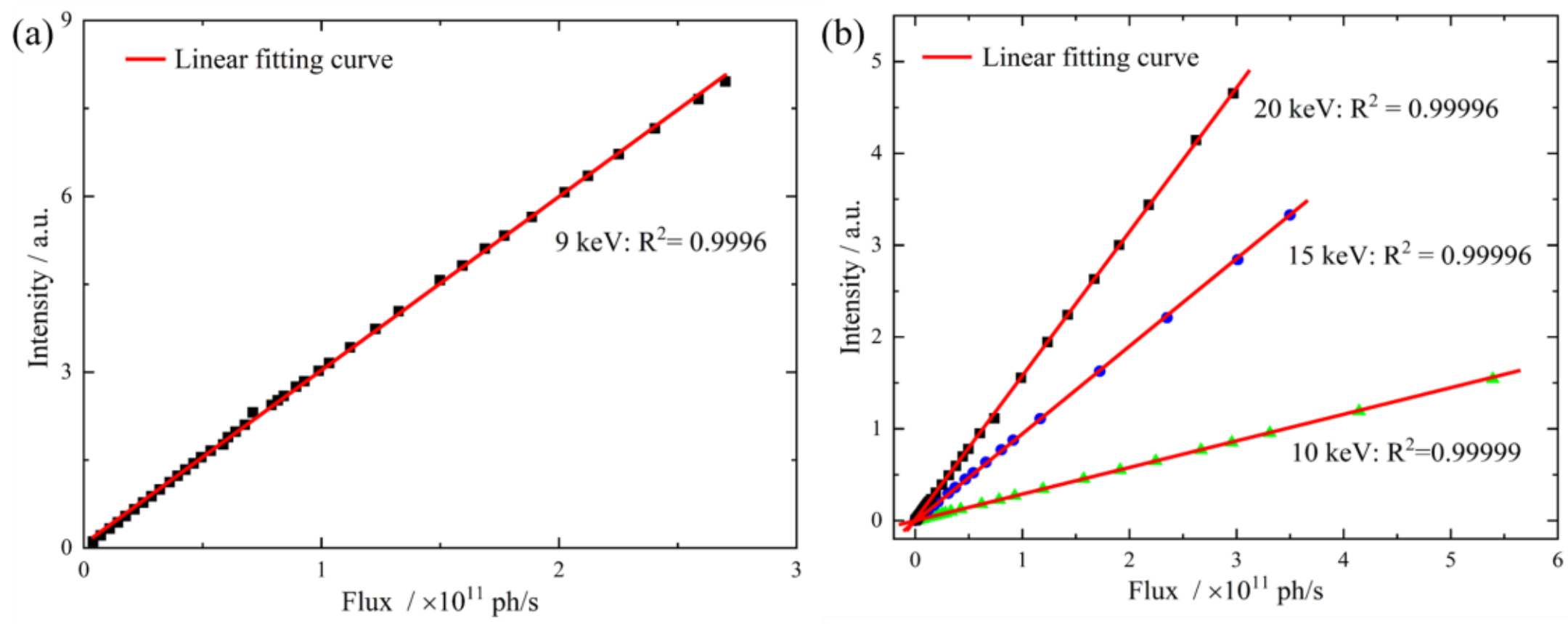

Figure S5 Linear-response evaluation of the integrating fluorescence detectors. Signals from (*a*) the fast-Lytle detector at 9 keV and (*b*) the PIPS detector at 10, 15 and 20 keV are plotted against the incident-flux monitor. A PTFE block was used to generate the scattering signal under a fixed measurement geometry. Solid red lines show linear least-squares fits.

Table S2 Summary of detectors for QXAFS measurement at BL11B beamline.

Detector	Gridded ionization chamber	Plate ionization chamber	PIPS	Fast-Lytle
Application scenario	transmission (I_0, I_t)		fluorescence (I_f)	
Typical signal strength	0.1–10 µA		10–1000 nA	0.1–10 nA
Typical amplifier gain (V/A)	$10^6 – 10^7$	$10^6 – 10^7$	$10^7 – 10^8$	$10^9 – 10^{10}$
Dimensions (mm)	Length: 170<br>Width: 140<br>Height: 100	Length: 200/350<br>Width: 100<br>Height: 100	Housing diameter: 30/60	Length: 120<br>Width: 113<br>Height: 113
Weight (kg)	6.6	2.8 (200 mm)<br>4.3 (350 mm)	0.16	3.6
Active area	-	-	φ28/ φ50 mm	90×90 mm^2
System response time[1]	< 26 µs	< 165 µs	< 30 µs	~ 650 µs
Linearity	Excellent			
Background Intensity	< 1 pA	< 1 pA	~ 20 pA	< 1 pA

Notes:

1. The system response time includes contributions from the intrinsic detector response, the rise time of the amplifier and the chopper speed.

S4. QXAFS control and synchronization system

For the rapid QXAFS measurements reported in this work, the data-acquisition software supplied by RI Research Instruments GmbH together with the QXAFS monochromator system was used. The hardware-level synchronization architecture is schematically illustrated in Fig. S6. The system utilizes a National Instruments PXIe-6366 DAQ board to simultaneously record the continuous analog voltage outputs from the fast current amplifiers (monitoring I_0, I_t, and I_f) and the digital pulse train from the Bragg angle encoder. An onboard master clock provides common-clock hardware timing for the encoder and detector channels, maintaining point-by-point correspondence between the measured Bragg angle and detector signals during rapid scanning.

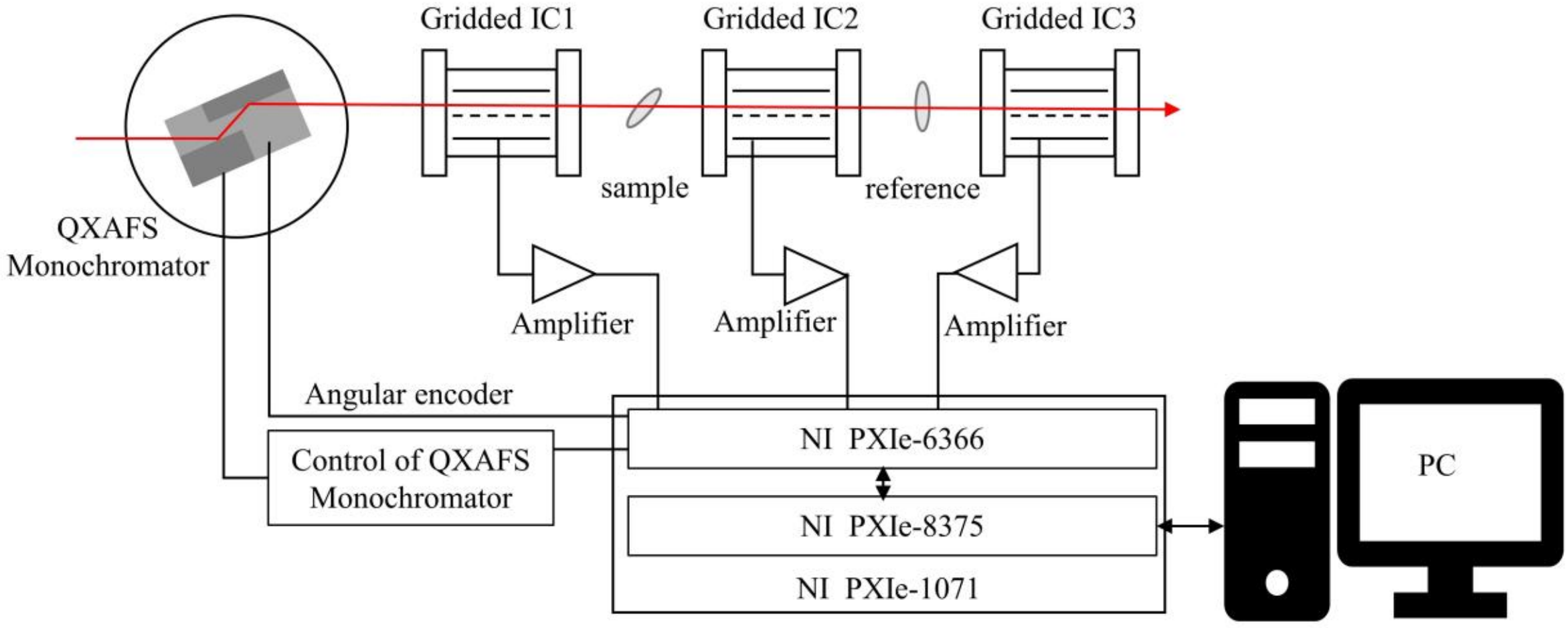

Figure S6 Schematic diagram of the hardware-level synchronization and control architecture for the QXAFS platform.

S5. Detailed user interface of QXAFS Data Studio

QXAFS Data Studio was developed as a stand-alone processing environment for local deployment at BL11B, building on the general processing workflow established in ProQEXAFS (Clark *et al.*, 2020), including angular binning, filtering, energy calibration and batch processing, while providing direct compatibility with the BL11B raw-data structure and PXIe-based synchronized acquisition system. Beamline-specific functions include automatic identification and separation of decreasing-angle (down) and increasing-angle (up) scans, management of long time-series datasets, configurable batch post-processing and optional application of a fixed direction-specific energy offset determined from a reference foil measured under identical monochromator conditions.

The "Import Data" module (Fig. S7) is designed for loading and previewing the synchronized encoder and detector data streams acquired at BL11B. The user selects the workflow mode (Standard or Advanced), specifies the absorber and edge, and maps the detector channels for transmission, reference or fluorescence processing. The software then parses the raw binary files, checks the common valid duration of the encoder and ADC streams, and automatically identifies the turning points of the Bragg-angle trajectory to segment the continuous acquisition into individual scans. In Standard mode, the sweep-boundary information is passed directly to the downstream processing workflow. In Advanced mode, an optional Feather export is available as an intermediate cache for inspection or debugging. Feather is a columnar Apache Arrow format that preserves numerical data types and enables rapid reloading without text parsing; it is therefore used only as an optional intermediate cache in Advanced mode.

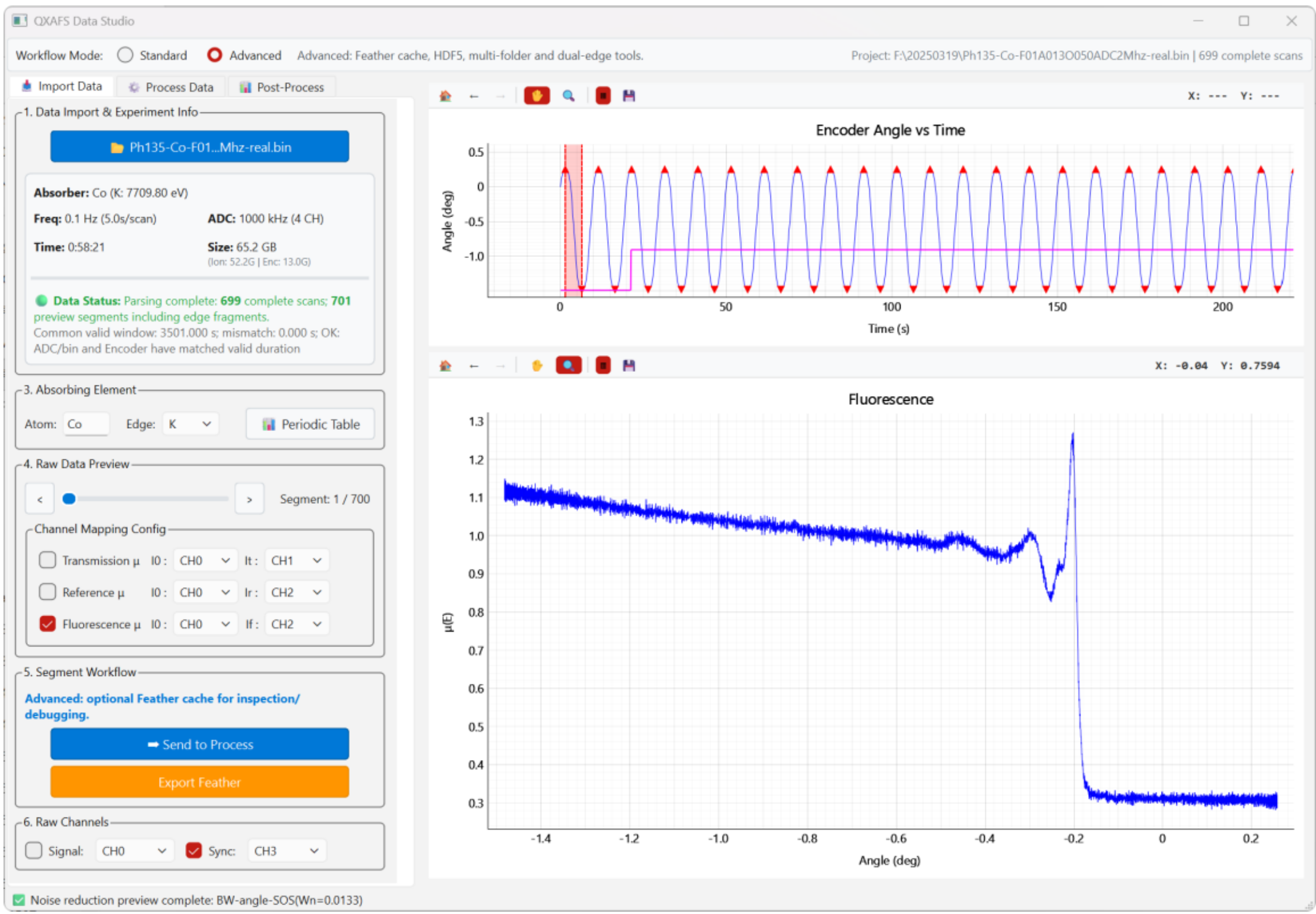

Figure S7 Graphical user interface of the "Import Data" module. The left panel provides controls for experiment information, absorbing-element selection, raw-data preview, channel mapping and segmentation workflow. The right visualization windows display the encoder-angle trajectory as a function of time (top) and the raw spectrum of the selected segment plotted against angle (bottom). The automatically detected turning points are marked on the encoder trace, and the Advanced mode optionally allows Feather export for inspection or debugging.

The "Process Data" module (Fig. S8) performs the main reduction of the segmented spectra. The selected segment source may be taken directly from the raw segmented stream or from an optional cached representation generated in Advanced mode. The module supports low-pass filtering after encoder-based angular binning and optional Savitzky-Golay smoothing. For energy calibration, the current implementation uses derivative-based edge finding, with the calibration angle previewed from the maximum of $-d\mu/d\theta$ within a user-defined angular interval. A fixed calibration position may also be supplied when a common reference angle is required for batch processing. Additional Advanced-mode tools, including adaptive EXAFS smoothing and dual-edge splitting, are provided for specialized datasets. After the parameters are established, the spectra can be batch-processed and exported in standard .dat format, with optional .h5 output.

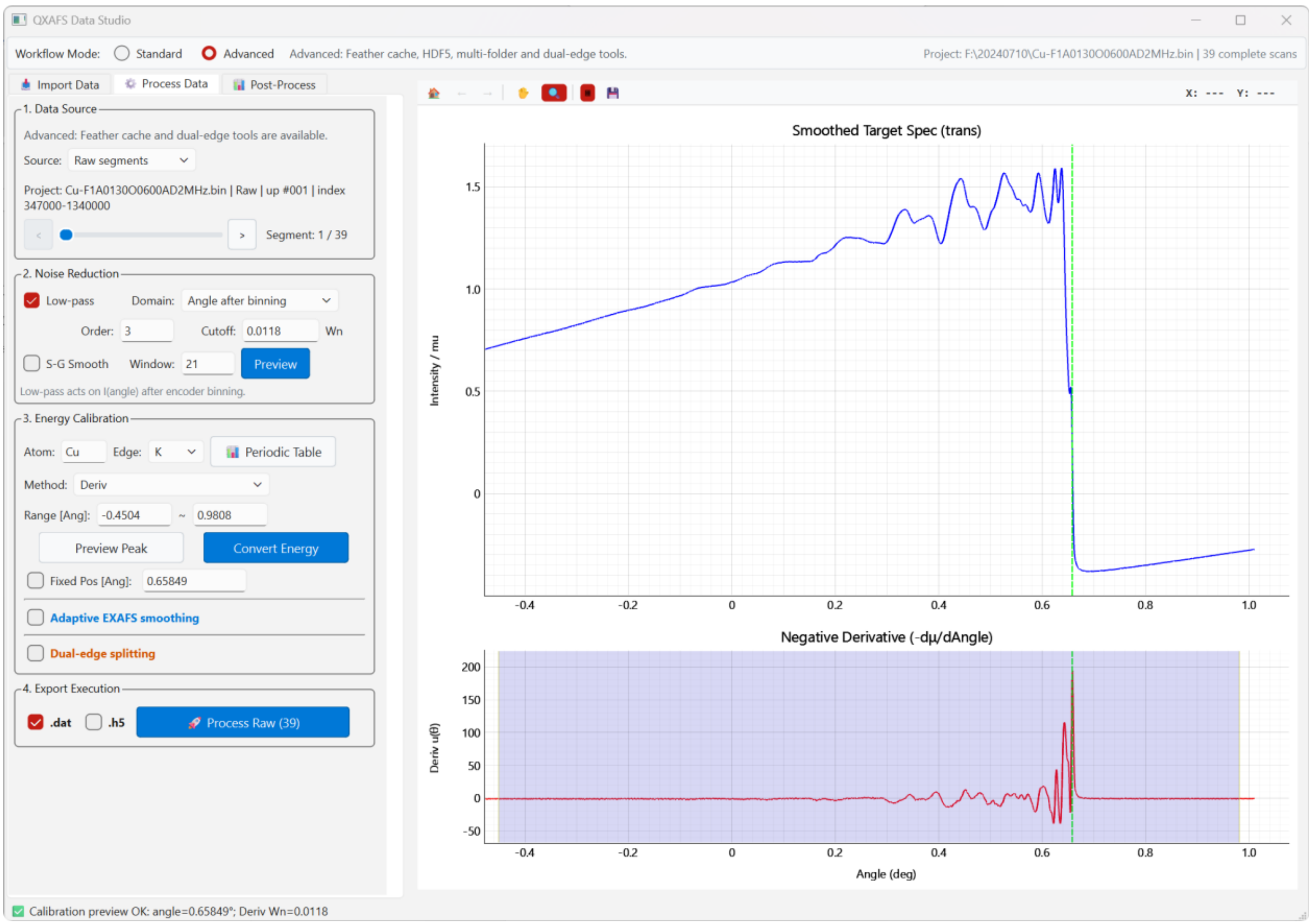

Figure S8 Graphical user interface of the "Process Data" module. The left panels provide controls for data source selection, noise reduction, derivative-based energy calibration and export execution. The right visualization windows show the filtered target spectrum (top) and the corresponding negative

derivative used to preview the calibration angle (bottom). The green dashed line marks the selected calibration position.

The "Post-Process" module (Fig. S9) is used for inspection and analysis of the exported spectra. A selected .dat file or spectrum series can be loaded for fluorescence, reference or merged-direction analysis. The module includes optional truncation of the energy range, an interactive scatter-deglitch tool, background normalization and standard transformations to *k*- and *R*-space. It also provides 3D visualization of XANES, χ(*k*) and |χ(*R*)| evolution across the spectrum series. For in situ measurements, kinetic descriptors such as edge position or edge shift can be tracked over the selected spectrum range, and a summary PDF report can be generated directly from the interface.

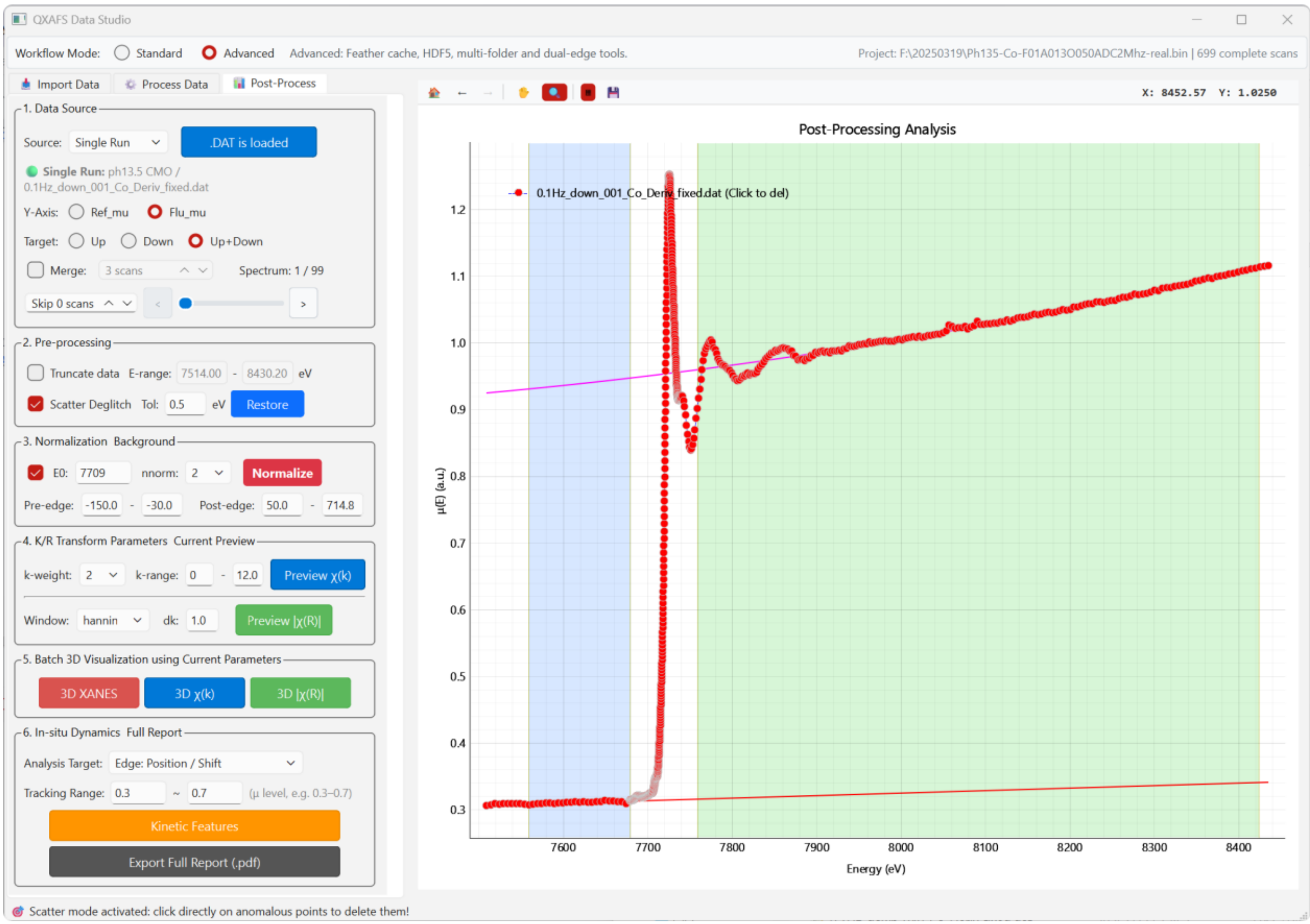

Figure S9 Graphical user interface of the "Post-Process" module. The left panels provide interactive controls for pre-processing, background normalization, *k*- and *R*-space transformations, 3D visualization and kinetic-feature extraction. The main visualization window displays the currently selected spectrum together with the user-defined pre- and post-edge regions used for normalization. The module is intended for rapid inspection of time-resolved datasets and for extraction of kinetic observables from large spectrum series.

S6. QXAFS data processing algorithms and data compression

For the QXAFS spectra presented in the platform-performance evaluation and *operando* case study, down and up scans were processed separately. Unless otherwise stated, each displayed QXAFS spectrum corresponds to an individual scan, and no averaging of consecutive spectra was applied. Figs. 6–8 present down-scan spectra, whereas Fig. 9 directly compares down- and up-scan spectra. The *operando* time series in Fig. 10 includes both scan directions. The angle-binned detector signals were processed using a third-order Butterworth low-pass filter. No machine-learning-based or additional post-processing denoising was applied. In contrast, the residual-based noise analysis described in Section 5 of the main manuscript and Section S8 of the Supporting Information was performed on rebinned raw absorption data without spectral filtering or normalization.

The raw quick-scanning X-ray absorption fine structure (QXAFS) data were acquired with a sampling rate of 2 MHz. For a QXAFS spectrum collected at a macroscopic oscillation frequency *f*, the initial number of data points per single energy scan is given by $10^6/f$ (i.e., 2 MHz/2*f*). To compress the dataset and improve the signal-to-noise ratio, the incident (I_0) and transmitted (I_t) intensities corresponding to identical Bragg angle encoder values were grouped and averaged (angular binning). The typical number of angle data points obtained after this initial compression at various oscillation frequencies is summarized in Table S3.

To suppress residual high-spatial-frequency fluctuations after encoder-position binning, a user-adjustable zero-phase Butterworth low-pass filter was applied. The filter function is defined as:

$$H(\omega) = \frac{1}{\sqrt{1 + \left(\frac{\omega}{\omega_c}\right)^{2N}}}$$

where ω is the normalized digital frequency, ω_c is the normalized cutoff frequency, and N is the filter order. Both the filter order N and normalized cutoff (W_n) can be modified by the user. A third-order design was used as the software default and for the datasets presented here. The filter coefficients were generated using *scipy.signal.butter,* and zero-phase filtering was performed using forward-backward filtering with *scipy.signal.filtfilt*. The W_n for the discrete filter was calculated as: $W_n = 2\Delta\theta/S_{ref}$, where $\Delta\theta$ is the angular sampling interval, and S_{ref} is an empirical reference angular scale. A value close to 0.003° was used as the default reference scale during the present data processing. This value is not a theoretically unique optimum; rather, it provides a practical means of obtaining dataset-specific normalized cutoff values while maintaining an approximately consistent angular cutoff scale.

The filtered intensities, $I_{0\_filtered}$ and $I_{t\_filtered}$, were used to calculate the absorption coefficient: $\mu = \ln(I_{0\_filtered}/I_{t\_filtered})$. A reference foil measured under the same monochromator conditions was used to determine the angular offset by assigning the maximum of dμ/dE for the reference spectrum to the tabulated edge energy. The QXAFS spectra were then interpolated onto a standardized energy

grid: 5 eV steps for the pre-edge region (-200 to -30 eV), 0.1 eV steps for the XANES region (-30 to 30 eV), and constant photoelectron wavenumber steps of Δk = 0.05 $Å^{-1}$ for the EXAFS region (>30 eV). Finally, the processed data were exported in the *.dat* format, ensuring compatibility with the Athena software for downstream analysis.

Table S3 Number of angle data points at different oscillation frequencies.

Oscillation frequency (Hz)	Angle data points (× 10^4)
0.5	~ 4.9
1	~ 4.9
10	~ 4.4
20	~ 3.8
30	~ 2.7
40	~ 1.8
50	~ 1.0

S7. Frequency- and direction-dependent spectral deviations in rapid QXAFS measurements

S7.1. Data processing and common energy-axis definition

For the analyses presented in this section, continuously acquired Cu foil spectra were first separated into decreasing-angle (down-scan) and increasing-angle (up-scan) branches using the turning points of the measured Bragg-angle encoder trajectory. Unless otherwise stated, the two scan directions were processed separately.

All spectra compared in Section S7 were reduced using the same angular-binning, filtering and fixed energy-calibration procedure. For each scan condition, the first processed Cu foil spectrum in the corresponding dataset was used to define the calibration angle by assigning the maximum of $d\mu/dE$ to the nominal Cu *K*-edge energy. The same calibration was then applied to all subsequent spectra acquired under that condition, without spectrum-by-spectrum energy realignment, so that any direction-dependent or scan-to-scan differences generated under a fixed calibration protocol are retained in the reconstructed spectra.

The effective edge position, E_0, was determined from the discrete maximum of $d\mu/dE$ for each processed spectrum and was used as a practical descriptor of reconstructed energy-axis reproducibility under rapid-scan conditions.

S7.2. Comparison of rapid-scan and conventional step-scan spectra

Fig. S10 compares the k^3-weighted Cu *K*-edge EXAFS spectra obtained by conventional step scanning and by rapid QXAFS under different oscillation frequencies. The principal EXAFS oscillations are retained in the rapid-scan spectra, confirming that the encoder-based energy reconstruction preserves the dominant structural information. Deviations and noise become more apparent mainly in the high-*k* region under the more demanding scan conditions.

This comparison indicates that the shortest demonstrated acquisition time is not necessarily the optimum condition for highest spectral fidelity over the full EXAFS range. Rather, the appropriate scan condition depends on the required balance between time resolution, scanned energy range and spectral quality.

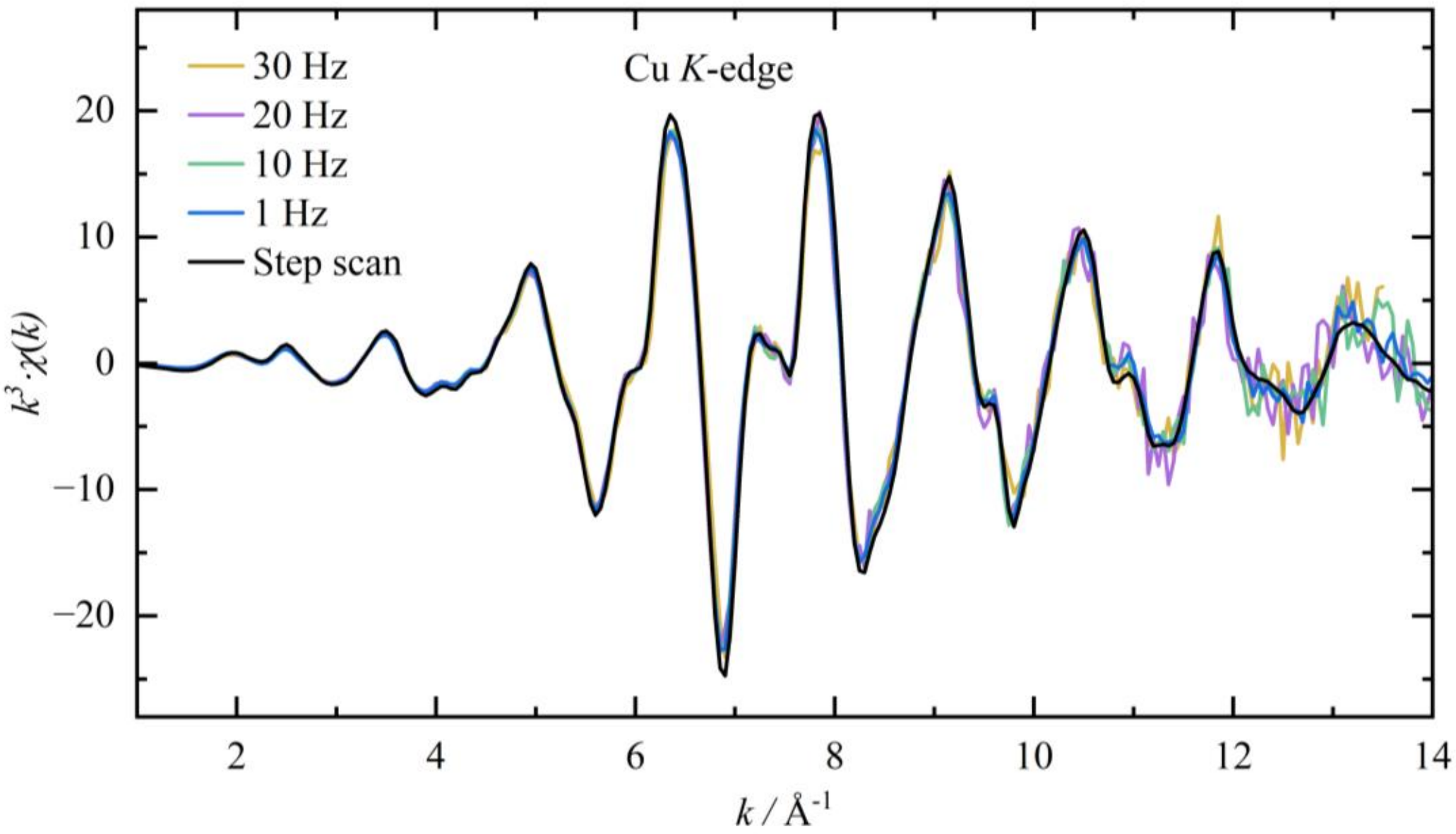

Figure S10 Comparison of k^3-weighted Cu *K*-edge EXAFS spectra obtained by conventional step scanning and rapid QXAFS measurements under different oscillation conditions.

S7.3. Direction-dependent edge-position statistics

Using the fixed-calibration procedure described in Section S7.1, the effective edge positions E_0 were determined for consecutive down- and up-scan spectra from the discrete maximum of dμ/dE.

Fig. S11 shows the re-centred distributions of E_0, plotted as

$$\Delta E_{0,i,f} = E_{0,i,f} - \text{median}\left(E_{0,\text{down},f}\right)$$

where the same constant shift was applied to both directions at a given frequency so that their relative separation was preserved. Table S4 summarizes the corresponding statistics from the original fixed-calibration results. The results show that:

1) the mean edge positions of the down- and up-scan branches are not identical;

2) finite scan-to-scan variability remains within each scan direction; and

3) neither the between-direction separation nor the within-direction spread changes monotonically with nominal oscillation frequency.

These observations indicate that the spectral differences are not limited to a simple constant offset between the two scan directions.

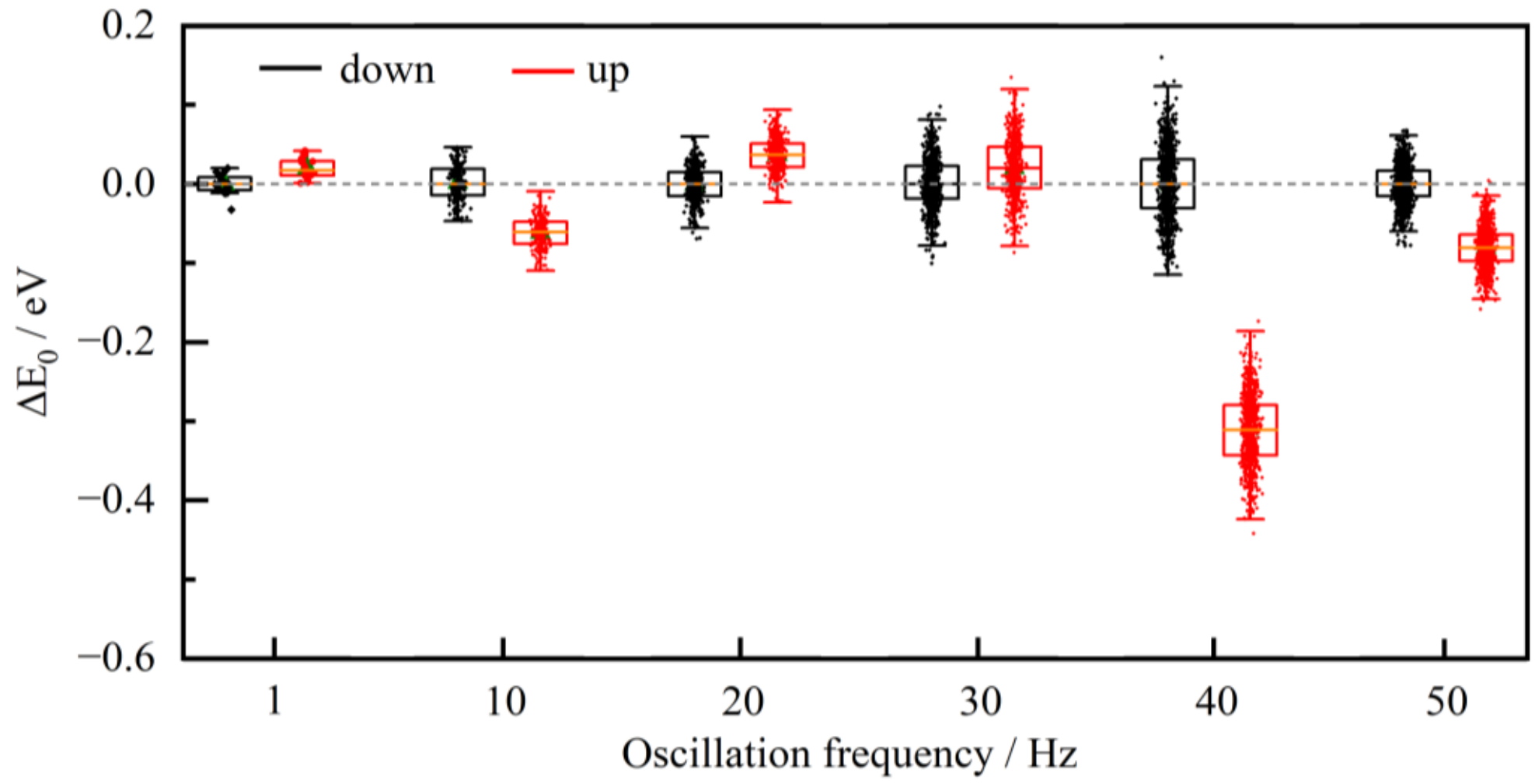

Figure S11 Re-centred distributions of the Cu *K*-edge positions obtained from down- and up-scan spectra at oscillation frequencies of 1, 10, 20, 30, 40 and 50 Hz. For each frequency, $\Delta E_0 = E_0 - \mathrm{median}(E_{0,\mathrm{down}})$, so that the median down-scan position was set to zero. The same constant shift was applied to both scan directions. Points represent individual spectra; boxes indicate the interquartile ranges, and central lines indicate the medians. Statistical results calculated from the original fixed-calibration data are summarized in Table S4.

Table S4 Statistical summary of the Cu *K*-edge positions obtained from consecutive down- and up-scan QXAFS spectra.

Frequency (Hz)	*N* (down/up)	SD (down/up) (eV)	R_{90} (down/up) (eV)	$\Delta\bar{E}_0$ (eV)
1	19/20	0.0125/0.0122	0.0315/0.0370	+0.0199
10	200/199	0.0219/0.0198	0.0709/0.0657	−0.0634
20	399/400	0.0219/0.0210	0.0711/0.0661	−0.1624
30	599/600	0.0326/0.0376	0.1073/0.1222	+0.0185
40	800/799	0.0447/0.0451	0.1474/0.1469	−0.3125
50	1000/999	0.0244/0.0250	0.0801/0.0827	−0.1279

Note: *N* is the number of spectra retained for each scan direction. SD is the sample standard deviation within one scan direction. R_{90} is defined as the width of the central 90% interval, $P_{95} - P_5$. The direction-dependent mean offset is defined as $\Delta\bar{E}_0 = \bar{E}_{0,\mathrm{up}} - \bar{E}_{0,\mathrm{down}}$.

S7.4. Measured scan trajectories and scan-rate distributions

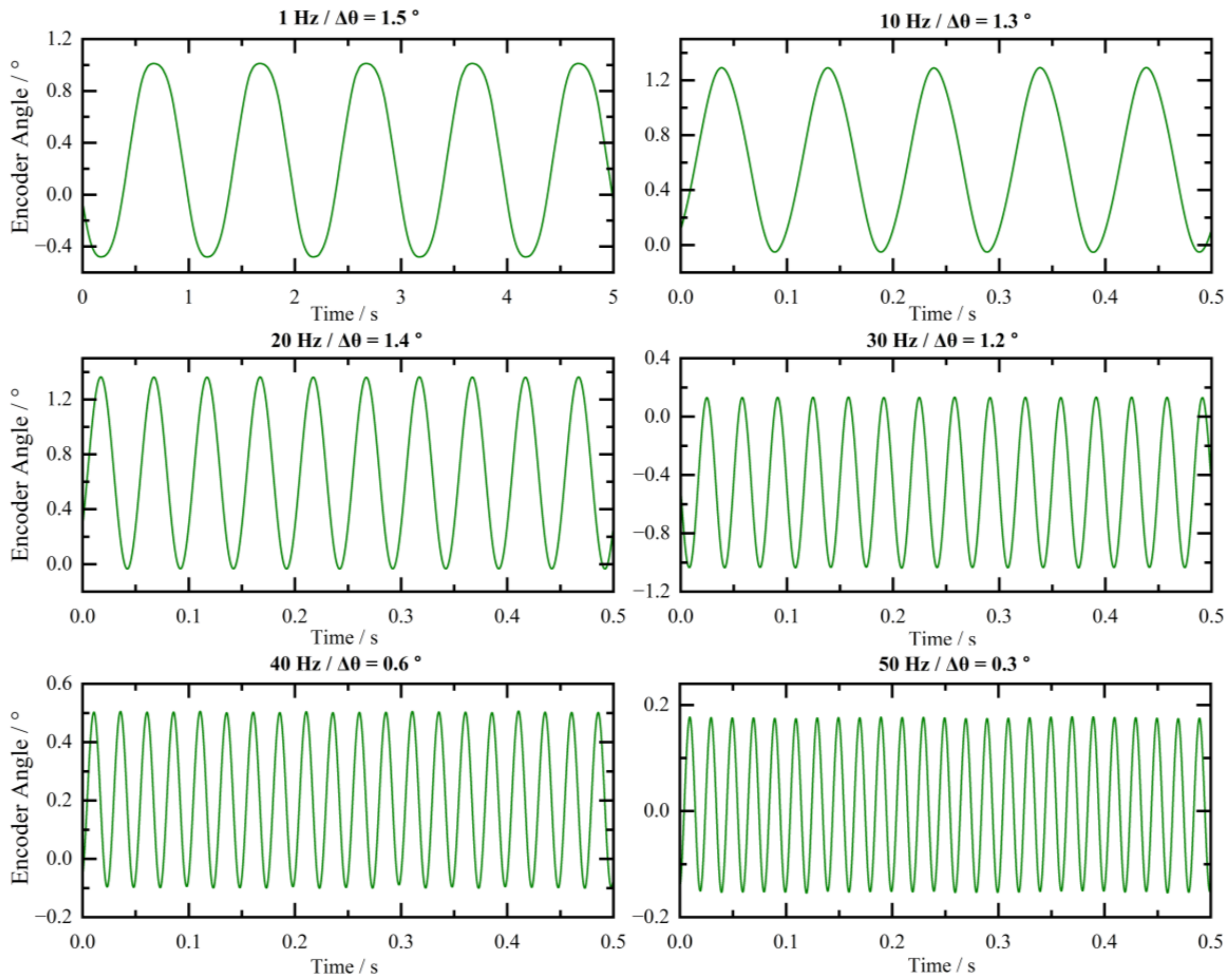

Figure S12 Measured Bragg-angle encoder trajectories for the investigated oscillation frequencies.

The nominal oscillation frequency alone does not uniquely determine the actual scanning condition. Fig. S12 compares the measured Bragg-angle encoder trajectories at different frequencies and shows that the angular oscillation range decreases at the highest-frequency settings.

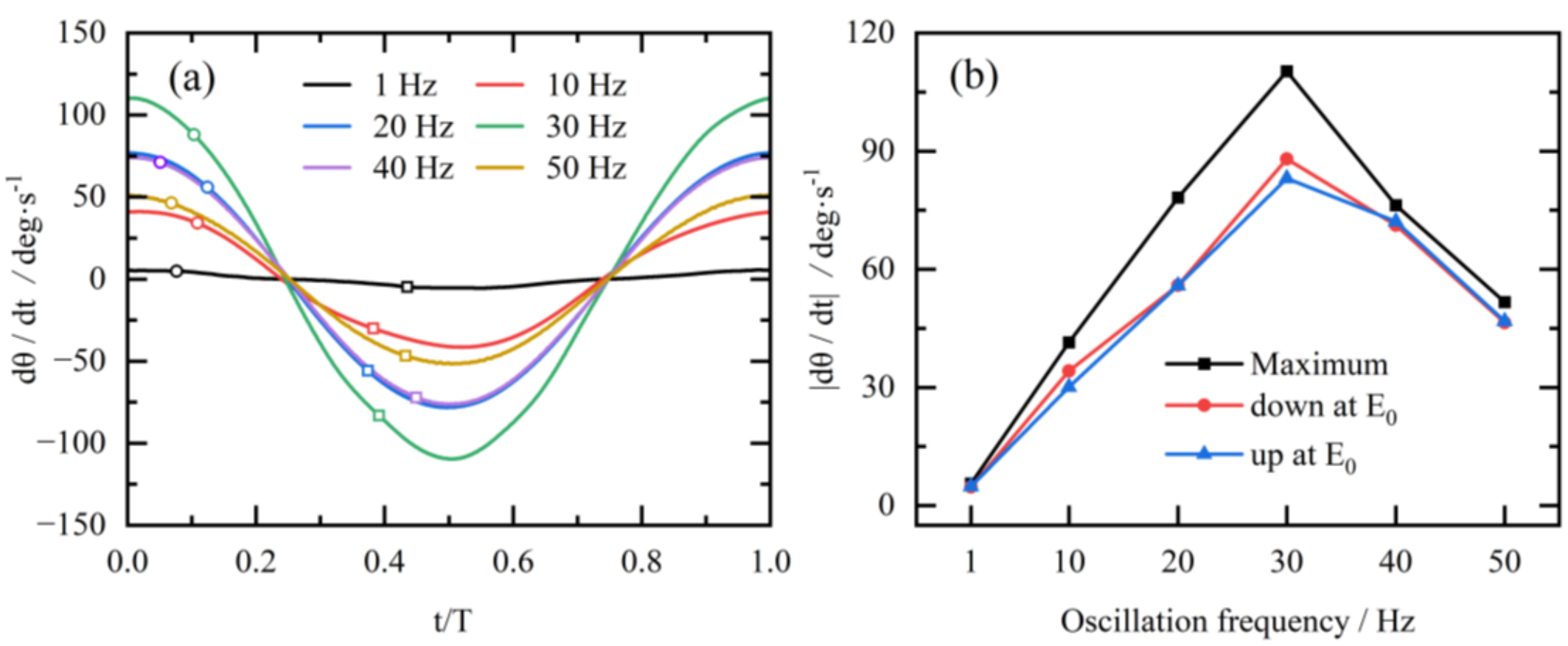

Figure S13 Encoder-derived angular velocities under different QXAFS operating conditions. (*a*) Angular-velocity profiles over one normalized oscillation cycle at 1, 10, 20, 30, 40 and 50 Hz. The open squares and circles indicate the velocities at the Cu *K*-edge for the down- and up-scan directions,

respectively. (*b*) Maximum angular-velocity magnitude within each cycle and local angular-velocity magnitudes at E_0 = 8979 eV.

The angular velocity, $\omega(t) = \mathrm{d}\theta(t)/\mathrm{d}t$, was calculated directly from the measured encoder signal. As shown in Fig. S13, the maximum angular velocity does not occur at the highest nominal frequency, but at an intermediate operating condition (f = 30 Hz) because the accessible angular range is reduced at 40–50 Hz.

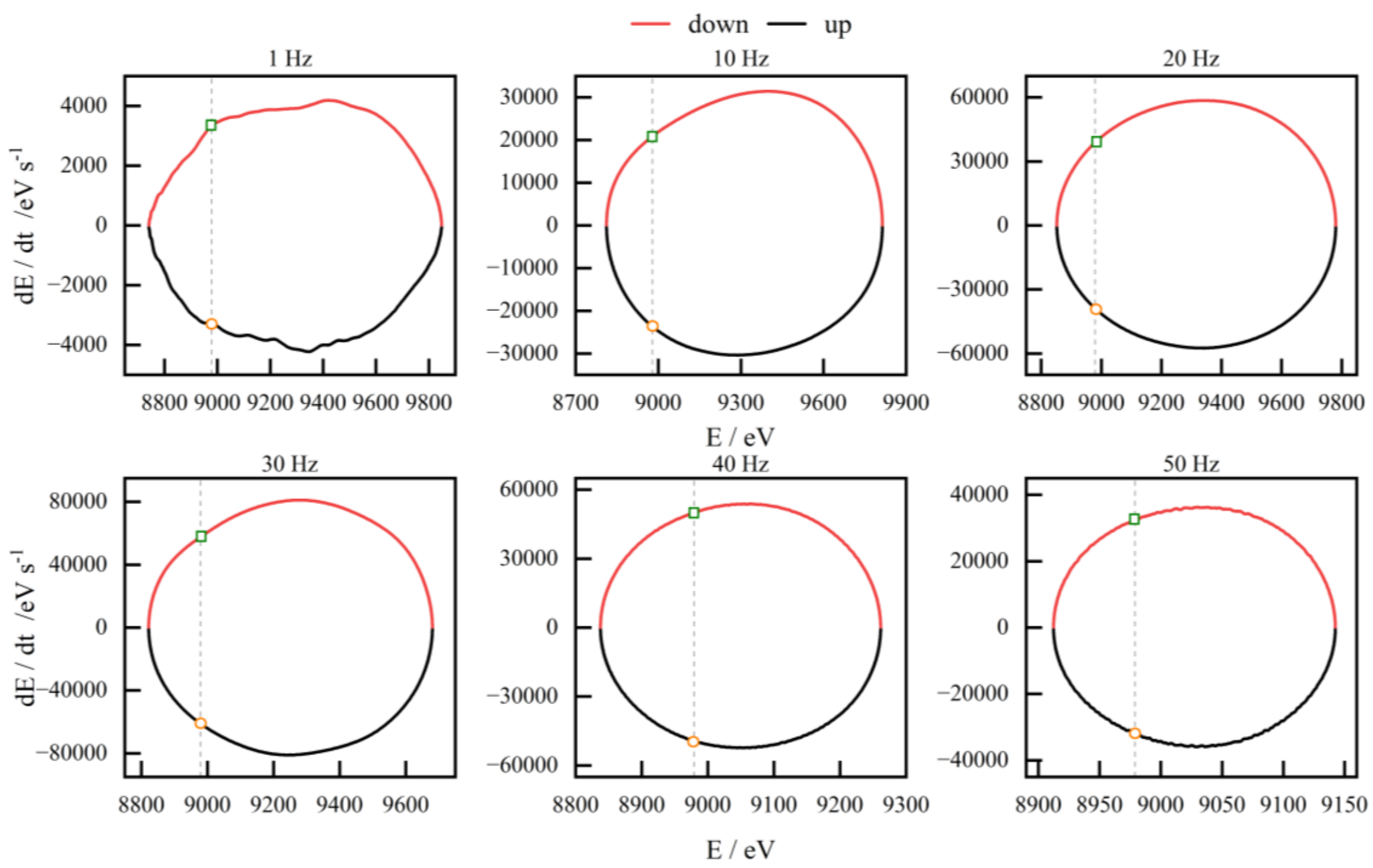

Figure S14 Instantaneous photon-energy scan rates calculated from the measured Bragg-angle trajectories. The red and black curves represent the down and up scans, respectively. Open squares and circles indicate E_0 = 8979 eV used to evaluate the local scan rates.

To express the monochromator motion directly in the spectral coordinate, the angular velocity was converted to the local energy scan rate according to $\mathrm{d}E(t)/\mathrm{d}t = [\mathrm{d}E(t)/\mathrm{d}\theta(t)] \times [\mathrm{d}\theta(t)/\mathrm{d}t] = \mathrm{E} \times \cot\theta(t) \times \omega(t)$. The corresponding instantaneous energy scan rate was obtained from the encoder-derived trajectory. Fig. S14 shows that the energy scan rate varies across each sweep and that its magnitude is also non-monotonic across the selected frequencies.

These results explain why the spectral deviations do not follow a simple monotonic dependence on nominal oscillation frequency alone. The effective scan condition is determined jointly by oscillation frequency, accessible angular range, actual trajectory and local energy scan rate.

S7.5. Interpretation and practical implications

Because the Bragg-angle encoder and detector channels were digitized synchronously, deviations of the actual crystal motion from the driving waveform are largely reflected in the reconstructed energy

axis. The observed direction-dependent spectral differences therefore cannot be assigned solely to an untracked mechanical lag. At the same time, the present measurements do not support reduction of the observed behaviour to a single detector-delay term.

Taken together, the statistical and kinematic results indicate that the residual deviations arise from the combined influence of the actual monochromator trajectory, the non-ideal response characteristics of the detection–readout chain, angular sampling and subsequent data processing. The present measurements establish the existence and practical magnitude of these effects, but do not permit the individual hardware contributions to be separated quantitatively. Accordingly, the analysis in this section should be interpreted as a practical assessment of reconstructed energy-scale reproducibility and spectral fidelity under rapid-scan operation, rather than as an independent determination of absolute monochromator energy stability.

For practical use, down- and up-scan spectra should be examined separately before averaging or direct comparison. If a stable reference foil is available and a reproducible direction-dependent rigid offset is observed under fixed scan conditions, an empirical direction-specific correction may be applied. Such a correction can compensate a stable constant offset, but it cannot recover information lost through line-shape distortion, smoothing or limited sampling.

S8. Quantitative residual-based noise analysis

The noise level of the Cu *K*-edge QXAFS spectra was quantitatively evaluated using a rigorous residual-based statistical method. To strictly avoid any numerical artifacts or signal smoothing introduced by data processing algorithms, the raw absorption data were analysed directly without applying any spectral filtering or normalization procedures.

The analysis was confined to the pre-edge region from 8859 to 8949 eV, corresponding to a Bragg-angle range of 12.8962°–12.7643°. This region contains no pronounced absorption features and therefore allows instrumental and statistical fluctuations to be evaluated with minimal contribution from genuine spectral structure. The 50 Hz dataset was excluded because its narrower scan window did not provide a sufficient pre-edge region within the common energy window.

To ensure statistical consistency across all scan frequencies, the oversampled raw data from a total of N_{total} consecutive spectra were rebinned onto a uniform angular grid. At higher oscillation frequencies, the discrete nature of sampling may result in empty bins within certain individual spectra. To address this, a "valid bin" criterion was implemented. The procedure was as follows:

1. The pre-edge angular window was divided into discrete uniform bins (index j).
2. For each bin, the number of spectra containing at least one valid data point was recorded as N_j. A bin was considered statistically valid only if its data retention reached at least 70% ($N_j/N_{\mathrm{total}} \geq 70\%$); otherwise, the bin was excluded from the noise calculation. Let M_{valid} denote the total number of such valid bins.
3. For a valid j-th bin (centred at angle θ_j), the local baseline reference $\bar{\mu}(\theta_j)$ was calculated by averaging the intensities of all N_j contributing data points:

$$\bar{\mu}(\theta_j) = \frac{1}{N_j}\sum_{i=1}^{N_j} \mu^i(\theta_j)$$

4. The deviation (pure residual) of each individual data point from its local bin average was extracted via direct subtraction:

$$\Delta\mu^i(\theta_j) = \mu^i(\theta_j) - \bar{\mu}(\theta_j)$$

5. The pre-edge residual noise magnitude (σ) was then quantified by calculating the root-mean-square (RMS) of the residuals across all M_{valid} bins that satisfied the 70% threshold:

$$\sigma_{\mathrm{pre-edge}} = \sqrt{\frac{1}{M_{\mathrm{valid}}}\sum_{j=1}^{M_{\mathrm{valid}}}\left\{\frac{1}{N_j - 1}\sum_{i=1}^{N_j}\left[\Delta\mu^i(\theta_j)\right]^2\right\}}$$

To evaluate the dependence of the residual noise on angular-bin size, N adjacent digitized encoder increments were combined into one analysis bin before calculating σ. Each encoder increment

corresponded to approximately 0.109 arcsec under the present acquisition conditions. Table S5 summarizes the resulting values for bin-merging factors from $N = 2$ to $N = 8$.

At a fixed bin-merging factor, σ increased from 0.5 to approximately 10 Hz and subsequently varied only weakly between 10 and 40 Hz. Increasing N reduced σ at all frequencies, although the reduction became progressively weaker at the higher scan rates. For statistically independent data, σ is expected to decrease approximately as $N^{-1/2}$, so that the scaled variance $\sigma^2 \cdot N$ remains approximately constant. The observed departure from this ideal scaling at high frequencies indicates increasing correlation between neighbouring angular bins. The corresponding scaled-variance analysis and its implications for detector-response-limited binning are discussed in Fig. 11(*b*) and Section 5 of the main manuscript.

Table S5 Dependence of the pre-edge residual noise magnitude σ on the monochromator oscillation frequency (f) and the bin-merging factor (N). The fundamental bin size ($N = 1$) corresponds to the digitized angular increments (~ 0.1 arcsec). To facilitate comparison, all noise values have been multiplied by 10^3.

Oscillation frequency (f)	Bin-merging factor (N)						
	2	3	4	5	6	7	8
0.5 Hz	1.43	1.20	1.06	0.97	0.89	0.83	0.78
1 Hz	1.88	1.63	1.44	1.33	1.23	1.14	1.08
10 Hz	3.41	3.02	2.64	2.40	2.27	2.22	2.18
20 Hz	3.51	3.43	3.31	3.18	3.03	2.89	2.72
30 Hz	3.43	3.37	3.29	3.19	3.09	2.98	2.87
40 Hz	3.41	3.32	3.22	3.10	2.97	2.85	2.71

References

Clark, A. H., Imbao, J., Frahm, R. & Nachtegaal, M. (2020). *J. Synchrotron Rad.* **27**, 551–557.

Lytle, F. W., Greegor, R. B., Sandstrom, D. R., Marques, E. C., Wong, J., Spiro, C. L., Huffman, G. P. & Huggins, F. E. (1984). *Nucl. Instrum. Methods Phys. Res., Sect. A*, **226**, 542–548.

Suzuki, M., Kawamura, N., Lytle, F. W. & Ishikawa, T. (2002). *J. Synchrotron Rad.* **9**, 99–102.
